# Development of antiseptic adaptation and cross-adapatation in selected oral pathogens in vitro

**DOI:** 10.1038/s41598-019-44822-y

**Published:** 2019-06-06

**Authors:** Tim Verspecht, Esteban Rodriguez Herrero, Ladan Khodaparast, Laleh Khodaparast, Nico Boon, Kristel Bernaerts, Marc Quirynen, Wim Teughels

**Affiliations:** 1 Department of Oral Health Sciences, University of Leuven (KU Leuven), Kapucijnenvoer 33, 3000 Leuven, Belgium; 2 Switch Laboratory, VIB Center for Brain and Disease Research, Herestraat 49, 3000 Leuven, Belgium; 3 Switch Laboratory, Department of Cellular and Molecular Medicine, University of Leuven (KU Leuven), Herestraat 49, 3000 Leuven, Belgium; 4 Center for Microbial Ecology and Technology (CMET), Ghent University (UGent), Coupure links 653, 9000 Gent, Belgium; 5 Bio- and Chemical Systems Technology, Reactor Engineering and Safety, Department of Chemical Engineering, University of Leuven (KU Leuven), Leuven Chem&Tech, Celestijnenlaan 200F (bus 2424), 3001 Leuven, Belgium; 6 Dentistry, University Hospitals Leuven, Kapucijnenvoer 33, 3000 Leuven, Belgium

**Keywords:** Bacteriology, Periodontitis, Antimicrobial resistance, Pathogens

## Abstract

There is evidence that pathogenic bacteria can adapt to antiseptics upon repeated exposure. More alarming is the concomitant increase in antibiotic resistance that has been described for some pathogens. Unfortunately, effects of adaptation and cross-adaptation are hardly known for oral pathogens, which are very frequently exposed to antiseptics. Therefore, this study aimed to determine the *in vitro* increase in minimum inhibitory concentrations (MICs) in oral pathogens after repeated exposure to chlorhexidine or cetylpyridinium chloride, to examine if (cross-)adaptation to antiseptics/antibiotics occurs, if (cross-)adaptation is reversible and what the potential underlying mechanisms are. When the pathogens were exposed to antiseptics, their MICs significantly increased. This increase was in general at least partially conserved after regrowth without antiseptics. Some of the adapted species also showed cross-adaptation, as shown by increased MICs of antibiotics and the other antiseptic. In most antiseptic-adapted bacteria, cell-surface hydrophobicity was increased and mass-spectrometry analysis revealed changes in expression of proteins involved in a wide range of functional domains. These *in vitro* data shows the adaptation and cross-adaptation of oral pathogens to antiseptics and antibiotics. This was related to changes in cell surface hydrophobicity and in expression of proteins involved in membrane transport, virulence, oxidative stress protection and metabolism.

## Introduction

Prevention and treatment of oral diseases such as tooth decay, gingivitis and periodontitis focusses primarily on removal of the dental plaque biofilm. The removal of dental plaque is frequently combined with the use of antimicrobials in mouth rinses and toothpastes such as chlorhexidine (CHX) and cetylpyridinium chloride (CPC)^1–3^. They are broad-spectrum cationic biocides, which generally affect the microbial membrane integrity of both Gram-positive and Gram-negative bacteria^4^. These antiseptics are also commonly used as co-adjuvants in the treatment of periodontal diseases such as gingivitis and chronic and aggressive periodontitis^5^, as the clinical anti-plaque efficacy of CHX and CPC on oral supragingival and subgingival biofilms has been shown extensively^1,6,7^.

Although these antiseptics are considered to be relatively safe, their unspecific mode of action and their long-term use via toothpastes and mouth rinses might create undesired and underestimated side effects. It has been documented that dead bacteria can stimulate the necrotrophic activity of periodontal pathogens, thereby increasing their growth and virulence^8^. Additionally, there is increasing awareness of the fact that the long-term, daily use of antiseptics can generate bacterial resistance due to the exposure to sub-lethal concentrations^9^. Some oral bacterial isolates can become resistant after being exposed to chlorhexidine. For instance, *Porphyromonas gingivalis* isolates increase their minimal inhibitory chlorhexidine concentration up to fourfold after exposure for 20 passages^10^. Moreover, *Enterococcus faecalis* increases its minimum inhibitory concentration (MIC) from 3 µg/mL to 11 µg/mL following 10 days of exposure to CHX^11^. Additionally, it is described that long-term use of antiseptics can also lead to increased MICs and thus resistance *in vivo*^5,12,13^.

This increase in MICs of antiseptics could potentially also result in resistance to antibiotics. Such cross-resistance phenotype is described for *Klebsiella*, *Proteus* and *Staphylococcus* species^14–16^. Acquired increased CHX resistance of *Klebsiella pneumoniae* was shown to result in cross-resistance to colistin, a last resort antibiotic^17^. Although a few studies already reported that some oral pathogens can become resistant to antiseptics after exposure, in general still little is known about this acquired antiseptic resistance/adapatation development and the concomitant changes at metabolic and protein levels.

It was hypothesized that repeated antiseptic exposure of the specific oral species used in this study would also result in increased antiseptic MICs and possibly in cross-resistance/adaptation to the other antiseptic and/or commonly used antibiotics. Furthermore, it was expected that this repeated exposure to antiseptics would also have an impact on the bacterial cell membrane and proteome.The aim of this study was to determine the effects of repeated exposure to CHX and CPC on six bacterial oral pathogens *in vitro*, namely *Aggregatibacter actinomycetemcomitans*, *Fusobacterium nucleatum*, *Porphyromonas gingivalis*, *Prevotella intermedia*, *Streptococcus mutans* and *Streptococcus sobrinus*. For several of these species, no or only limited knowledge about the impact of antiseptic exposure is available in terms of (cross-)resistance/adaptation development and proteomic alterations. In a first phase, this was done by monitoring the increase in antiseptic MICs of these pathogens during several passages in presence of antiseptics. Furthermore, given the mode of action of CHX and CPC, it was examined whether cross-adaptation to the other antiseptic and/or antibiotics commonly used in oral healthcare could occur. We hypothesized that (cross-)adaptation effects are accompanied by changes in cell surface properties and at protein level. The latter was investigated for the first time for these species under these specific conditions by mass spectrometry.

## Results

### Antiseptic adapatation development

**Figure 1:**
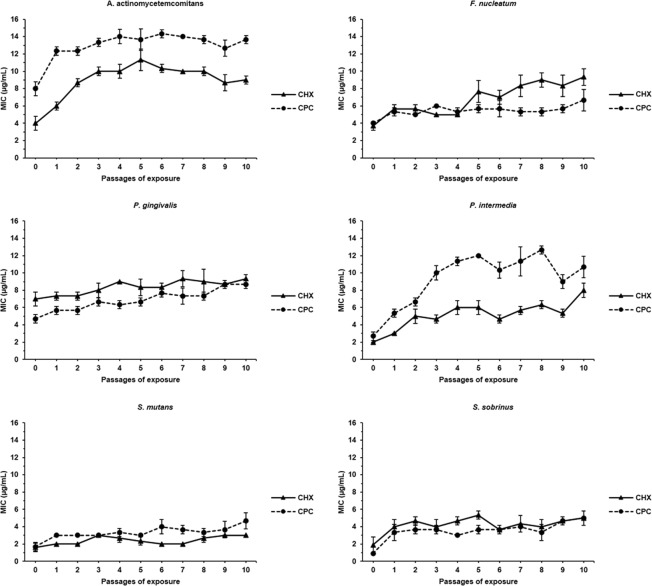
Monitoring of MIC values of CHX and CPC against six oral pathogens. Each oral pathogen was exposed to chlorhexidine (CHX) and cetylpyridinium chloride (CPC) during 10 consecutive passages. Minimum inhibitory concentrations (MICs) of both antiseptics against each pathogen were determined after each passage of exposure. Passage 0 corresponds to the unexposed wild type bacteria. Data are shown as average MIC values ± SD (n = 3).

**Figure 2:**
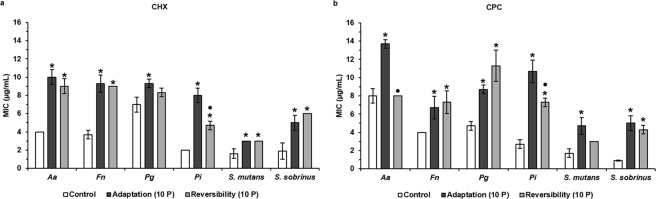
Evaluation of antiseptic adaptation and its reversibility by determination of MIC values of CHX and CPC against six oral pathogens. *Aa*: *A. actinomycetemcomitans*; *Fn*: *F. nucleatum*; *Pg*: *P. gingivalis; Pi*: *P. intermedia*; MIC: Minimum Inhibitory Concentration; CHX: chlorhexidine; CPC: cetylpyridinium chloride; Control: unexposed wild type bacteria; Adaptation (10 P): bacteria exposed to CHX (**a**) or CPC (**b**) during 10 passages; Reversibility (10 P): bacteria exposed to CHX (**a**) or CPC (**b**) during 10 passages and regrown in absence of antiseptics during 10 passages. Data are shown as average MIC values ± SD (n = 3). Statistically significantly higher MIC values when compared to the wild type control are marked with ‘*’ (*P* < 0.05), statistically significant differences between ‘Adaptation (10 P)’ and ‘Reversibility (10 P)’ are marked with ‘•’ (*P* < 0.05).

The adaptation of oral pathogens to CPC and CHX was monitored during exposure to these antiseptics for 10 consecutive passages. For each passage, bacteria were regrown in presence of one antiseptic concentration below the MIC observed for the previous passage. A tendency for adaptation, as observed by increasing MIC values, for all bacterial species and for both antiseptics was observed along the passages compared to the unexposed wild type strains (Fig. 1). At the 10^th^ passage, the increase in CHX and CPC MIC values ranged between 1.3- and 2.5-fold for all the pathogens, except for *P. intermedia* that increased its MICs for CHX and CPC four-fold and for *S. sobrinus* that increased its MIC for CPC almost six-fold (Fig. 2, Supplementary Table S1). Based on these results, the 10-passages CHX- and CPC-exposed bacteria were further investigated in the following experiments and were referred to as CHX- or CPC-adapted bacteria.

### Antiseptic and antibiotic cross-adaptation development

**Figure 3:**
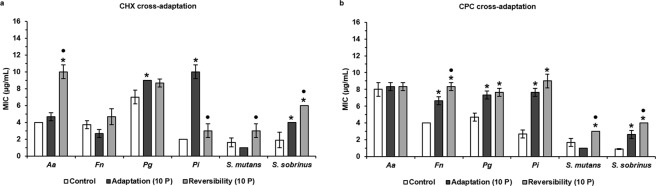
Evaluation of antiseptic cross-adaptation and its reversibility by determination of MIC values of CHX and CPC against six oral pathogens. (**a**) MIC values of CHX against bacteria initially exposed to CPC. (**b**) MIC values of CPC against bacteria initially exposed to CHX. *Aa*: *A. actinomycetemcomitans*; *Fn*: *F. nucleatum*; *Pg*: *P. gingivalis; Pi*: *P. intermedia*; MIC: Minimum Inhibitory Concentration; CHX: chlorhexidine; CPC: cetylpyridinium chloride; Control: unexposed wild type bacteria; Adaptation (10 P): bacteria exposed to CPC (**a**) or CHX (**b**) during 10 passages; Reversibility (10 P): bacteria initially exposed to CPC (**a**) or CHX (**b**) during 10 passages and regrown in absence of antiseptics during 10 passages. Data are shown as average MIC values ± SD (n = 3). Statistically significantly higher MIC values when compared to the wild type control are marked with ‘*’ (*P* < 0.05), statistically significant differences between ‘Adaptation (10 P)’ and ‘Reversibility (10 P)’ are marked with ‘•’ (*P* < 0.05).

To verify if the antiseptic-adapted bacteria concomitantly show an increase in MIC of other antiseptics or antibiotics, the MIC values for the other antiseptic (CPC for the CXH-adapted bacteria and CHX for the CPC-adapted bacteria) and for four commonly used antibiotics (amoxicillin, azithromycin, metronidazole, tetracycline) were determined for wild type and antiseptic-adapted bacteria. Certain CHX- and CPC-adapted oral pathogens became concomitantly more adapted to CPC and CHX, respectively, when compared to the wild type bacteria (Fig. 3, Supplementary Table S2). CHX-adapted *F. nucleatum*, *P. gingivalis*, *P. intermedia* and *S. sobrinus* significantly increased their MICs for CPC 1.7-, 1.6-, 3.7- and 3-fold, respectively. In addition, CPC-adapted *P. gingivalis*, *P. intermedia* and *S. sobrinus* significantly increased their MICs for CHX 1.3-, 3.9- and 2.1-fold, respectively.

**Figure 4:**
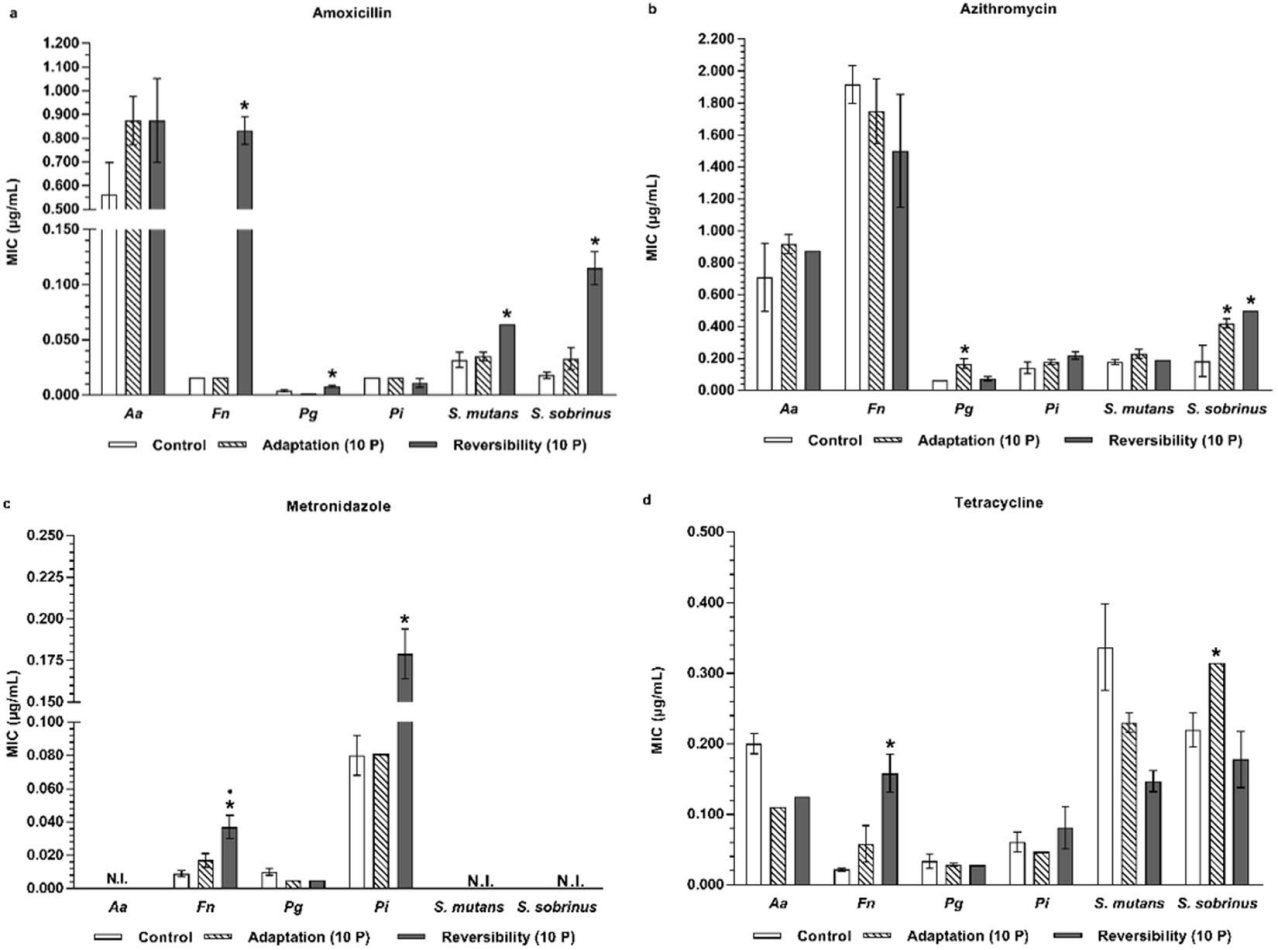
Evaluation of antibiotic cross-adaptation and its reversibility by MIC determination of four commonly used antibiotics against six oral pathogens exposed to CHX. (**a**) MIC values of amoxicillin against CHX-exposed bacteria. (**b**) MIC values of azithromycin against CHX-exposed bacteria. (**c**) MIC values of metronidazole against CHX-exposed bacteria. (**d**) MIC values of tetracycline against CHX-exposed bacteria. *Aa*: *A. actinomycetemcomitans*; *Fn*: *F. nucleatum*; *Pg*: *P. gingivalis; Pi*: *P. intermedia*; MIC: Minimum Inhibitory Concentration; CHX: chlorhexidine; Control: unexposed wild type bacteria; N.I.: not inhibited; Adaptation (10 P): bacteria exposed to CHX during 10 passages; Reversibility (10 P): bacteria exposed to CHX during 10 passages and regrown in absence of antiseptics during 10 passages. Data are shown as average MIC values ± SD (n = 3). Statistically significantly higher MIC values when compared to the wild type control are marked with ‘*’ (*P* < 0.05). Statistically significantly different MIC values when comparing ‘Reversibility (10 P)’ to ‘Adaptation (10 P)’ are marked with ‘^**•**^’ (*P* < 0.05).

**Figure 5:**
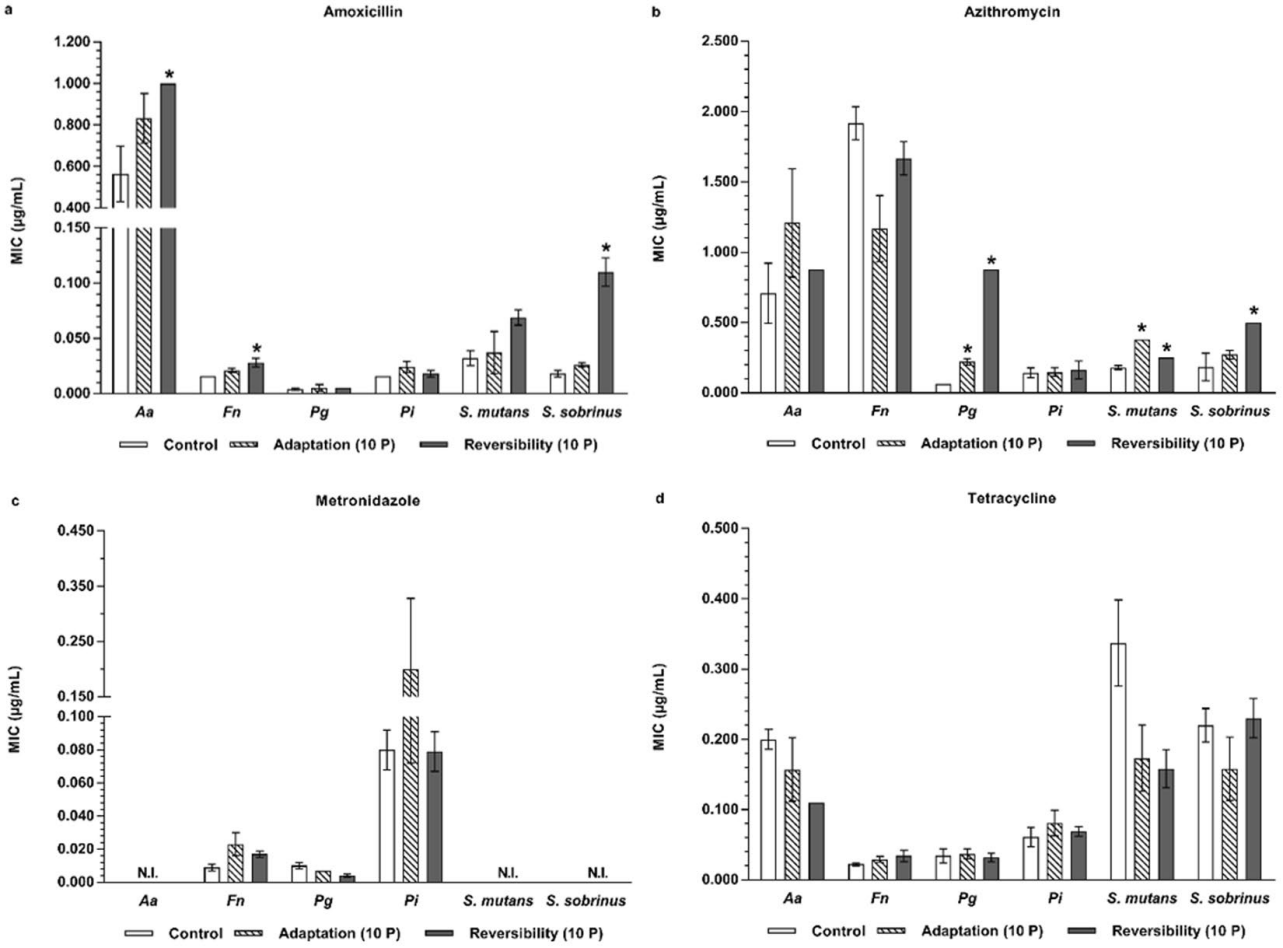
Evaluation of antibiotic cross-adaptation and its reversibility by MIC determination of four commonly used antibiotics against six oral pathogens exposed to CPC. (**a**) MIC values of amoxicillin against CPC-exposed bacteria. (**b**) MIC values of azithromycin against CPC-exposed bacteria. (**c**) MIC values of metronidazole against CPC-exposed bacteria. (**d**) MIC values of tetracycline against CPC-exposed bacteria. *Aa*: *A. actinomycetemcomitans*; *Fn*: *F. nucleatum*; *Pg*: *P. gingivalis; Pi*: *P. intermedia*; MIC: Minimum Inhibitory Concentration; CPC: cetylpyridinium chloride; Control: unexposed wild type bacteria; N.I.: not inhibited; Adaptation (10 P): bacteria exposed to CPC during 10 passages; Reversibility (10 P): bacteria exposed to CPC during 10 passages and regrown in absence of antiseptics during 10 passages. Data are shown as average MIC values ± SD (n = 3). Statistically significantly higher MIC values when compared to the wild type control are marked with ‘*’ (*P* < 0.05). Statistically significantly different MIC values when comparing ‘Reversibility (10 P)’ to ‘Adaptation (10 P)’ are marked with ‘^**•**^’ (*P* < 0.05).

A similar cross-adaptation development to antibiotics was observed for some antiseptic-adapted species when compared to the wild type controls (Figs 4, 5, Supplementary Tables S3, S4). Increased MICs were observed for all antiseptic-adapted species for azithromycin, except for the antiseptic-adapted *F. nucleatum*, which showed a decreased MIC for this antibiotic. However, only for CHX-adapted *P. gingivalis* and *S. sobrinus* and CPC-adapted *P. gingivalis* and *S. mutans*, this increase could reach the level of significance. For the other antibiotics, a significantly increased MIC for tetracycline was observed for the CHX-adapted *S. sobrinus*.

### Reversibility of the antiseptic and antibiotic (cross-)adaptation effects

To elucidate if the increased MICs and bacterial (cross-)adaptation to antiseptics and antibiotics are (partially) reversible, CHX- and CPC-adapted bacteria were regrown in absence of antiseptics for 10 passages. MIC determinations showed that all CHX- and CPC-adapted species regrown in the absence of antiseptics, except for CHX-adapted *P. gingivalis* and CPC-adapted *A. actinomycetemcomitans* and *S. mutans*, had significantly increased antiseptic MICs compared to their wild type controls (Fig. 2, Supplementary Table S1). The level of antiseptic MICs remained in general similar between the antiseptic-adapted and the regrown antiseptic-adapted species with the exception of CHX-adapted *P. intermedia* and CPC-adapted *A. actinomycetemcomitans*, for which a significant decrease in MICs was noted when regrown in the absence of the antiseptics. Although both significantly decreased their MICs compared to the antiseptic-adapted species, CHX-adapted *P. gingivalis* still showed a significantly higher MIC compared to the wild type control, whereas the MIC of CPC-adapted *A. actinomycetemcomitans* dropped back to the same MIC value as the wild type control. In the case of CPC-adapted *P. gingivalis*, even a significant increase in MIC was observed after regrowth in absence of the antiseptic. Therefore, antiseptic adaptation appeared to be at least partially conserved in most of the pathogens.

Possible loss of the cross-adaptation to antiseptics was evaluated identically. Most of the antiseptic-adapted bacteria that showed cross-adaptation to the other antiseptic maintained their cross-adaptation and thus their elevated MICs for this antiseptic (Fig. 3, Supplementary Table S2). However, CPC-adapted *P. gingivalis* and *P. intermedia* lost their cross-adaptation to CHX. Surprisingly, CHX-adapted *S. mutans* and CPC-adapted *A. actinomycetemcomitans* showed cross-adaptation to CPC and CHX, respectively, after regrowth in absence of antiseptics. Furthermore, in the case of CPC-adapted *S. sobrinus* and CHX-adapted *F. nucleatum* and *S. sobrinus*, their antiseptic MICs even increased further (*P* < 0.05) after regrowth in absence of antiseptics.

In a similar approach, the possible loss of the cross-adaptation for the CHX- and CPC-adapted species to antibiotics was evaluated (Figs 4, 5 and Supplementary Tables S3, S4). From the CHX-adapted bacteria, only *S. sobrinus* maintained its cross-adaptation to azithromycin and from the CPC-adapted bacteria, only *P. gingivalis* maintained and even significantly increased its elevated azithromycin MIC. Conversely, CHX-adapted *P. gingivalis* and *S. sobrinus* lost their increased azithromycin and tetracycline MICs, respectively. Also CPC-adapted *S. mutans* showed a decrease in its cross-adaptation to azithromycin. Similar to what was observed for antiseptic cross-adaptation reversibility, several antiseptic-adapted bacterial species actually increased their antibiotic MICs when regrown for 10 passages in the absence of the antiseptic. For instance, CHX-adapted *F. nucleatum* acquired a 52.1-fold higher amoxicillin MIC, a 2.18-fold higher metronidazole MIC and a 2.72-fold higher tetracycline MIC after 10 passages without antiseptics (Fig. 4, Supplementary Table S3), although the wild type species exposed to CHX during 10 passages did not show cross-adaptation to amoxicillin. Similar observations of acquisition of antibiotic cross-adaptation were made for CHX-adapted *P. gingivalis* (amoxicillin, 4-fold increase in MIC), *P. intermedia* (metronidazole, 2.21-fold increase in MIC), *S. mutans* (amoxicillin, 1.83-fold increase in MIC), *S. sobrinus* (amoxicillin, 3.48-fold increase in MIC) and for CPC-adapted *S. sobrinus* (amoxicillin and azithromycin, 4.23-fold and 1.32-fold increase in MIC, respectively) repassaged in absence of antiseptics.

### Changes in cell surface hydrophobicity

**Figure 6:**
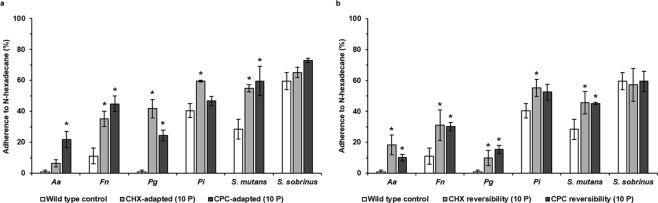
Cell surface hydrophobicity analysis of wild type and antiseptic-adapted oral pathogens. (**a**) Cell surface hydrophobicity of antiseptic-adapted bacteria. Wild type control: unexposed wild type bacteria; CHX-adapted (10 P): bacteria adapted to chlorhexidine (CHX) after exposure during 10 passages; CPC-adapted (10 P): bacteria adapted to cetylpyridinium chloride (CPC) after exposure during 10 passages. (**b**) Cell surface hydrophobicity of antiseptic-adapted bacteria regrown in absence of antiseptics. Wild type control: unexposed wild type bacteria; CHX reversibility (10 P): bacteria initially exposed to CHX during 10 passages and regrown in absence of antiseptics during 10 passages; CPC reversibility (10 P): bacteria initially exposed to CHX during 10 passages and regrown in absence of antiseptics during 10 passages*. Aa*: *A. actinomycetemcomitans*, *Fn: F. nucleatum*, *Pg*: *P. gingivalis*, *Pi*: *P. intermedia*. Cell surface hydrophobicity is represented as the average percentage of adherence to N-hexadecane ± SD (n = 3). Statistically significantly different values when compared to the wild type control are marked with an ‘*’ (P < 0.05).

In order to verify if changes in cell-surface properties of the oral pathogens could be one of the possible underlying mechanisms explaining the observed effects, the cell-surface hydrophobicity was determined. All antiseptic-adapted species had a higher cell surface hydrophobicity, compared to their wild type controls (Fig. 6a). This increased cell surface hydrophobicity reached a level of statistical significance for CHX-adapted *F. nucleatum, P. gingivalis, P. intermedia and S. mutans* and CPC-adapted *A. actinomycetemcomitans, F. nucleatum*, *P. gingivalis* and *S. mutans*. Cell surface hydrophobicity of antiseptic-adapted species regrown in absence of antiseptics for 10 passages was determined as well. For these strains as well, all bacteria had a higher cell surface hydrophobicity compared to their wild type controls, except for *S. sobrinus*, which showed similar values (Fig. 6b). These increases were statistically significant for CHX-adapted *A. actinomycetemcomitans*, *F. nucleatum*, *P. gingivalis*, *P. intermedia* and *S. mutans* regrown in absence of CHX and for CPC-adapted *A. actinomycetemcomitans*, *F. nucleatum*, *P. gingivalis* and *S. mutans* regrown in absence of CPC.

### Proteomic analysis of antiseptic-adapted species

**Figure 7:**
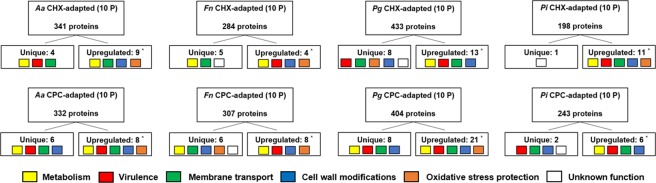
Overview of the proteomic analysis of antiseptic-adapted species in terms of unique or upregulated proteins. *Aa*: *A. actinomycetemcomitans*; *Fn*: *F. nucleatum*; *Pg*: *P. gingivalis*; *Pi*: *P. intermedia*; CHX: chlorhexidine; CPC: cetylpyridinium chloride; CHX-adapted (10 P): bacteria exposed to CHX during 10 passages; CPC-adapted (10 P): bacteria exposed to CPC during 10 passages. Unique proteins were not detected in the wild type species; upregulated proteins were significantly increased compared to the wild type species (*, *P* < 0.05). Colored squares represent the main functional domains associated with the detected proteins.

**Figure 8:**
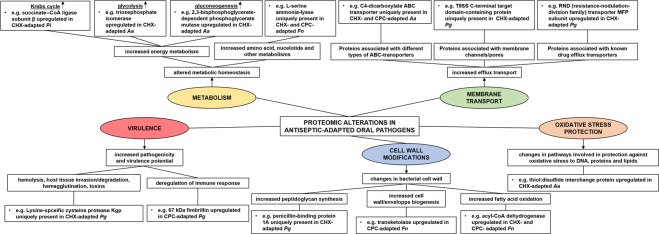
General overview of the main proteomic alterations in antiseptic-adapted oral pathogens. Proteomic analysis of antiseptic-adapted oral pathogens by means of mass spectrometry revealed alterations in protein expression compared to the unexposed wild type species. When looking at the functions of the uniquely present or upregulated proteins, they appeared to be mainly involved in bacterial metabolism, membrane transport, cell wall modifications, bacterial virulence and oxidative stress protection. More specifically, exposure of oral pathogens to antiseptics resulted in proteomic changes that can possibly be associated with an altered metabolic homeostasis (increased energy, amino acid, nucleotide and other metabolisms), increased efflux transport, changes in bacterial cell wall, increased bacterial pathogenicity and virulence, and changes in pathways involved in protection against oxidative stress to DNA, proteins and lipids. Uniquely present proteins were not detected in the wild type species; upregulated proteins were significantly increased compared to the wild type species A more detailed overview of all proteins and more examples can be found in Supplementary Tables S5–S9. *Aa*: *A. actinomycetemcomitans*; *Fn*: *F. nucelatum*; *Pg*: *P. gingivalis*; *Pi*: *P. intermedia*; CHX: chlorhexidine; CPC: cetylpyridinium chloride. CHX-adapted species: bacteria exposed to CHX during 10 passages. CPC-adapted species: bacteria exposed to CPC during 10 passages.

Since the periodontopathogens acquired the highest MIC levels and showed the largest changes in cell-surface hydrophobicity, proteomic analysis was performed for the CHX- and CPC-adapted periodontopathogens and their wild type controls. For all antiseptic-adapted periodontal species, clear alterations in a wide range of bacterial metabolic and behavioral pathways following the exposure to antiseptics were shown (Fig. 7, Supplementary Tables S5–S8). These changes at protein level were most pronounced in CHX- and CPC-adapted *P. gingivalis*, both in terms of upregulation and unique presence of certain proteins compared to the wild type control. In contrast, *P. intermedia* showed the least changes at protein level when considering the numbers of upregulated or unique proteins together. Additionally, differences in the amount of upregulated or unique proteins were observed depending on the type of antiseptic that was used. Exposure to CPC generally induced higher numbers of upregulated or unique proteins than exposure to CHX. When looking at functions associated with the uniquely present or upregulated proteins, they appeared to be mainly involved in bacterial metabolism, membrane and cell wall modifications, membrane transport, bacterial virulence and resistance to oxidative stress (Figs 7, 8, Supplementary Tables S5–S8). It is noteworthy that there were also a considerable number of proteins downregulated in the antiseptic-adapted species, or only detected in the unexposed wild type species. An overview of these particular proteins can be found in Supplementary Table S9.

## Discussion

This *in vitro* study showed that oral pathogens can develop adaptation to CHX and CPC upon exposure to sub-inhibitory concentrations of these antiseptics, which lead to statistically significantly increased MICs. Concomitantly, the occurrence of cross-adaptation to other antiseptics (CPC or CHX) and antibiotics was shown. These increased MICs are (partially) maintained after discontinuing the exposure to the antiseptic. Additionally, it was shown that wild type and antiseptic-adapted bacterial strains grown under identical conditions differ in cell surface hydrophobicity and in unique and/or upregulated expression of proteins involved in bacterial metabolism, membrane and cell wall modifications, membrane transport, bacterial virulence and resistance to oxidative stress. As far as we know, this study is the first one to examine the impact of antiseptic exposure *in vitro* on these six different species at the same time and to investigate the accompanying effects at protein level through mass spectrometry analysis.

The exposure of oral pathogens to CHX or CPC resulted in a progressive increase in the antiseptic MICs over the different passages, with the 10^th^ passage generally yielding the highest MIC levels. All pathogens increased their MICs with factors ranging from 1.3 to 5.5, depending on the bacterial species and the antiseptic. These data are in line with other studies, where some of these species also showed increases in MICs for CHX in the same order of magnitude. For instance, some clinical oral isolates of *P. gingivalis* increased their MIC values for CHX up to fourfold after 20 passages in the presence of sub-inhibitory concentrations of CHX^10^. In addition, the oral pathogen *Enterococcus faecalis* was also found to increase its MICs for CHX up to 3.6-fold after 10 passages, but not for CPC^11^. Also *Streptococcus sanguinis* strains have been shown to increase their CHX MICs 8-fold^18^. Although two recent studies did not show a significant increase in *S. mutans* CHX and/or CPC MICs after repeated exposure^10,11^, the current data shows the opposite. Since the test conditions in one of the two latter studies were quite similar to those described in this study, it therefore can be hypothesized that antiseptic resistance/adaptation development is not only species-dependent, but also strain-dependent. Indeed, strain differences in antiseptic resistance development have been shown for *Staphylococcus aureus* and *P. gingivalis* strains^10,19^. The observed *in vitro* CHX adaptation for *A. actinomycetemcomitans*, *F. nucleatum* and *P. intermedia* and CPC adaptation for the oral pathogens tested in this study has never been reported yet and/or deeply studied. However, in regards to the latter, *Pseudomonas stutzeri*, *Pseudomonas aeruginosa* and *Campylobacter jejuni* have shown adaptation to CPC^20,21^. The clinical relevance of these *in vitro* data is unclear, since there is a general conception that CHX and CPC do not induce resistance *in vivo*, and are therefore safe^13,22–27^. However, the studies on which these conceptions are based are not always properly designed, are not including the bacterial species of interest, are showing an increase in MICs or are even lacking in the case of CPC. Schiott and coworkers (1976) did show increased CHX MICs in the total salivary flora and for selected salivary streptococci after 17 and 23 months of CHX use^28^. Similar observations were made by Emilson and Fornell (1976) following a 1-year CHX treatment^12^. Also, Maynard and coworkers (1993) reported an increase in MIC for anaerobic supragingival plaque bacteria for CHX during a 6-month trial^13^. However, a triple application of a CHX gel or varnish in a 1-week period did not change the CHX MIC of salivary *S. mutans* and *S. sobrinus* in a period of 4 to 28 weeks after application^29^. Unfortunately, none of these studies looked at specific bacterial species which we nowadays link with periodontal diseases. Our *in vitro* data support these rudimentary clinical findings. However, it should be taken into account that there are distinct differences between our *in vitro* study and the real-life situation in the daily practice. Firstly, the exposure time used in the current study (*i.e*. 24 h) is different from usual exposure times in real-life situations (*i.e*. 30 s - 1 min, 3 times a day). Following on that, the current study looked at which changes occurred after 10 passages of exposure. This in contrast with the real-life situation, where people often use mouth rinses on a daily basis for weeks, months or even years. Secondly, there is a difference in terms of antiseptic concentrations. In our study, the antiseptic concentrations used in the MIC experiments ranged from 0.1–21 µg/mL, yielding MICs in the ranges of 3–10 µg/mL (CHX) and 3–13.7 µg/mL (CPC). This is considerably different from antiseptic concentrations commonly found in commercial mouth rinses, for instance 0.12–0.2% CHX (~1200–2000 µg/mL) or 0.07% CPC (~700 µg/mL). Lastly, also other factors could play important roles *in vivo*, such as dilution and clearance of antiseptics by the saliva.

In order to determine the possible reversibility of the induced antiseptic adaptation, the pathogens were repassaged in absence of CHX or CPC. The results showed that the induced adaptation was usually at least partially retained in absence of the antiseptic. *S. sanguinis*, *P. stutzeri* and *P. aeruginosa* show a similar (partial) behavior for CHX and CPC^18,21^. The *in vitro* data are in contrast with the findings of Schiott *et al*. (1976) who showed a reversal to control MIC values for CHX after 3 to 6 months^28^. However, in this study, no differentiation was made between different bacterial species and the students included in this study did not suffer from periodontal diseases or tooth decay. Therefore, one cannot be sure that after discontinuing an antiseptic therapy or during a prolonged antiseptic therapy, increased MIC levels will return to pre-treatment levels, or will not keep rising.

Since the early 2000’s, several studies have already shown that frequent usage of antiseptics can be accompanied by increased MICs and even resistance to antibiotics^16,30,31^. Repeated exposure of human bacterial pathogens to biocides can indeed act as a driver of antibiotic resistance development^32^. Our data are in line with this concept. Increased antibiotic MIC values were recorded for the CHX- or CPC-adapted strains albeit these increases were at maximum twofold. Interestingly, for some antiseptic-adapted strains that were cultured for 10 passages without the antiseptic, MICs for the tested antibiotics even increased more. A similar observation was made by Tattawasart and coworkers (1999) for Pseudomonas species^21^. At the moment it is unclear what might explain this increase in MICs. In general, the increased MIC values were still below the achievable salivary concentrations of the respective antibiotics^33^. However, the antiseptic-induced increase in *A. actinomycetemcomitans* MICs for amoxicillin brought its MIC value above the salivary amoxicillin concentration of 0.7 µg/mL.

Since CHX and CPC are both cationic biocides, they share their main mode of action. Both act as positively charged surfactants that bind to the negatively charged phospholipids of the bacterial cell membranes, which eventually leads to rupture, cytoplasmic leakage and cell death^15,34,35^. Therefore, it was hypothesized that antiseptic-adapted bacteria could possibly become cross-adapted to the other antiseptic. Three out of six of both the CHX- and CPC-adapted species showed such cross-adaptation. In line with the mode of action of both antiseptics, one could expect that the antiseptic-adapted bacteria displayed changes in their cell membranes compared to the unexposed wild type controls. For both the CHX- and CPC-adapted pathogens, four out of the six species significantly increased their surface hydrophobicity. For the same antiseptic-adapted pathogens regrown in absence of antiseptics, this was the case for five (CHX) and four (CPC) out of the six species. This can possibly be an underlying mechanism of the observed adaptation in the pathogens. It is known that biocide resistance often occurs through changes in membrane permeability and properties^21,36^. However, as not all six CHX- and CPC-adapted species showed an increase in surface hydrophobicity, and since in another study *E. faecalis* exposed to CHX and CPC only became resistant to CHX, but increased its hydrophobicity in both cases of exposure^11^, one can expect that other mechanisms play a role. In addition, it is noteworthy that 4 out of the 6 bacterial species used in this study are Gram-negatives (*A. actinomycetemcomitans*, *F. nucleatum*, *P. gingivalis, P. intermedia)*, whereas the other 2 species (*S. mutans* and *S. sobrinus*) are Gram-positives. Given the differences in membrane structure between both types of bacteria, such as the absence of an outer membrane for Gram-positives, one could maybe anticipate major differences in terms of hydrophobicity changes. However, this was not the case, as both the Gram-negative and Gram-positive species increased their hydrophobicity. As described above, both antiseptics act by binding to negatively charged components. This is associated with the cytoplasmic membrane itself (present in both Gram-positives and Gram-negatives), polysaccharide elements and teichoic acids (Gram-positives) and lipopolysaccharides (outer membrane of Gram-negatives)^37^. Compared to the antiseptic-adapted species, the majority of antiseptic-adapted species regrown in absence of antiseptics showed similar trends (with respect to their observed changes in MIC values) in cell surface hydrophobicity changes compared to their wild type controls. However, CHX-adapted *A. actinomycetemcomitans* regrown in absence of CHX, which had a similar CHX MIC compared to the same antiseptic-adapted species, significantly increased its cell surface hydrophobicity whereas this was not the case for CHX-adapted *A. actinomycetemcomitans*. Similarly, CPC-adapted *P. gingivalis* regrown in absence of CPC, which had a higher CPC MIC compared to the same antiseptic-adapted species, had a lower cell surface hydrophobicity compared to CPC-adapted *P. gingivalis*. Nevertheless, there was still a statistically significant increase in cell surface hydrophobicity compared to the wild type control in both cases. All this supports the hypothesis that changes in cell surface hydrophobicity are possibly (in part) one of the underlying mechanisms of the antiseptic adaptation observations made, or at least one of their accompanying effects. However, based on these results, one cannot simply draw a direct causal relationship between changes in cell surface hydrophobicity and the observed adaptation effects.

To investigate other potential adaptation mechanisms and (accompanying) effects, the protein fractions of CHX- and CPC-adapted periodontal bacteria, as well as those of the corresponding unexposed wild type bacteria, were analyzed by means of mass spectrophotometry. The analysis of the proteomes of the antiseptic-adapted species revealed alterations in specific survival and adaptive mechanisms because of the exposure to antiseptics. The proteomics data showed that antiseptic-adapted bacteria changed their metabolic profiles for instance by the upregulation of proteins involved in energy metabolism and more specifically in glycolysis, gluconeogenesis, citric acid cycle and lysine degradation pathways. Additionally, levels of proteins involved in amino acid, nucleotide and inorganic ion metabolisms were also increased. Similar changes have already been described in multi-resistant *Staphylococcus aureus* and *Enterococcus* species resistant to important antibiotics such as methicillin, vancomycin, linezolid, and daptomycin^38^. Proteomic analyses of these antibiotic-resistant species showed an increase in the presence of glyceraldehyde-3-phosphate dehydrogenase, 2,3-bisphosphoglycerate-dependent phosphoglycerate mutase, alcohol dehydrogenase and succinyl-CoA synthetase, which all were found to be also upregulated in the antiseptic-adapted periodontal species^39–42^. This might indicate that certain bacterial metabolic adaptations are conserved in response to exposure to antiseptics as well as to antibiotics, suggesting that these proteins could be specific targets in the prevention of bacterial resistance. In addition, antiseptic-adapted periodontal species showed proteomic differences possibly related to defense mechanisms against the antiseptics. For instance, we observed the upregulation and/or unique presence of proteins associated with several types of ABC transporters, which have already been related to the efflux of antibiotics in several multi-resistant species^43^. Furthermore, CHX-adapted *P*. gingivalis significantly upregulated its RND transporter MFP subunit. Transcriptional upregulation of efflux transporters belonging to the resistance-nodulation-division (RND) family of efflux transporters is something that was also observed in CHX-exposed *Burkholderia cenocepacia*, a Gram-negative bacterium involved in cystic fibrosis pathogenesis for which highly CHX-resistant strains have been described^44,45^. Additionally, other underlying mechanisms could possibly be found in the upregulation of several proteins related to membrane and cell wall modifications that are, more specifically, associated with increased synthesis of peptidoglycan and lysine, cell envelop biogenesis and β-oxidation of fatty acids. Similar alterations in cell wall architecture have been reported in non-oral bacteria resistant to antimicrobial compounds^38,46,47^.

Furthermore, the proteomic analysis of the antiseptic-adapted species revealed the upregulation of proteins involved in resistance to oxidative stress damage to proteins, lipids and DNA, caused by the use of antiseptics and antibiotics^48^. Resistance to oxidative stress is often also related to bacterial resistance to antibiotics^49^. These proteins have also been found to be upregulated in non-oral species resistant to multiple antibiotics^38,50^.

The exposure to CHX and CPC also induced the upregulation and the unique presence of virulence-related proteins in periodontal pathogens, suggesting that antiseptics can trigger an increase in pathogenicity and virulence potential of these pathogens. For instance, gingipain proteins (RgpA and Kgp), which are arginine or lysine specific cysteine proteinases produced by *P. gingivalis*, were only detected in CHX- and CPC-adapted *P. gingivalis*. These proteins are involved in hemolysis and iron uptake, degradation of host tissues, deregulation of the immune response and bacterial co-aggregation^51^. Other virulence proteins such as hemagglutinin A, hemolysin secretion protein D and tRNA(Glu)-specific nuclease (WapA), which are related to hemagglutination, hemolysis and toxin activity, were also either upregulated or uniquely present in the antiseptic-adapted species. In contrast with this effect, a study on the exposure of *S. aureus* to sub-lethal vancomycin concentrations found decreased amounts of most proteins with a virulence-related function^52^. However, in another study, the proteomic analysis of methicillin-resistant *S. aureus* revealed the unique presence of two virulence-related proteins, fibrinogen-binding protein and bone sialoprotein-binding protein, which are involved in attachment to the host tissue and play a key role in the initiation of endovascular infections and prosthetic-device infections^41^.

Future studies should further elucidate the underlying mechanisms and look at clinically more relevant situations. Firstly, sequencing of the different types of bacteria obtained throughout this study might be interesting to reveal possible genomic changes that in turn could be linked to the observed effects and changes at protein level. Given the unexpected results in the reversibility experiments, proteomic analysis of the bacteria obtained in these experiments could shed a new light on this complex phenomenon. Furthermore, different strains and/or clinical isolates of the bacterial species used in this study could be subjected to similar experiments, in combination with the use of antiseptic concentrations and exposure times/frequencies mimicking more real-life situations. This could provide more insights into the clinical significance and relevance of the obtained results.

In conclusion, six oral pathogens were found to become adapted to antiseptics after repeated exposure to CHX and CPC. This antiseptic adaptation was in general at least partially conserved after regrowth in absence of antiseptics. Furthermore, some of the antiseptic-adapted species also showed cross-adaptation to antibiotics and the other antiseptic. The possible underlying mechanisms are changes in cell surface hydrophobicity and alterations at the proteomic level. Moreover, most antiseptic-adapted species increased their cell surface hydrophobicity and (over)expressed proteins with functions that can be related to the observed (cross-)adaptation effects.

## Materials and Methods

### Bacterial species, media and antiseptics

*Prevotella intermedia* ATCC 25611, *Porphyromonas gingivalis* ATCC 33277, *Fusobacterium nucleatum* DSM 20482, *Streptococcus mutans* ATCC 20523, *Streptococcus sobrinus* ATCC 20742 and *Aggregatibacter actinomycetemcomitans* ATCC 43718 were maintained on blood agar (Blood agar Base I, Oxoid, Basingstoke, UK) supplemented with hemin (Sigma-Aldrich, St. Louis, MO, USA) (5.0 mg/mL), menadione (Calbiochem-Novabiochem, La Jolla, USA) (1.0 mg/mL) and 5% sterile horse blood (E&O Laboratories, Bonnybridge, Scotland). Broth cultures were prepared in Brain Hearth Infusion (BHI) broth (Difco, Detroit, USA). Minimum Inhibitory Concentration (MIC) experiments were performed in Brain Hearth Infusion (BHI) containing chlorhexidine (CHX) and cetylpyridinium chloride (CPC) (Sigma-Aldrich, St. Louis, USA). Development of adapted species was performed in BHI or on blood plates supplemented with CHX or CPC, depending on the bacterial strain. For all experiments, bacteria were cultured under aerobic (5% CO_2_) or anaerobic (80% N_2_, 10% H_2_ and 10% CO_2_) conditions. Optical densities were measured at 600 nm (OD_600_) by spectrophotometry using a GeneQuant spectrophotometer (Biochrom Ltd., Cambridge, United Kingdom).

### Antiseptic adaptation development

Overnight cultures of *A. actinomycetemcomitans*, *F. nucleatum*, *S. mutans* and *S. sobrinus* were adjusted to 10^7^ CFU/mL (OD_600_ ~ 0.5). CHX or CPC was dissolved in BHI at final concentrations ranging from 0.1 to 21 µg/mL and resulting solutions were added to a 96-well plate together with the adjusted bacterial cultures (final OD_600_ ~ 0.250), followed by a 24-h incubation at 37 °C under anaerobic conditions. MICs were determined by visual examination as well as by optical measurement at 600 nm by means of a Multiskan Ascent 96-well plate reader (Thermo Fisher Scientific, Waltham, USA). The MIC value was defined as the minimum antiseptic concentration at which no bacterial growth could be observed.

Bacterial solutions from the last well in which bacterial growth could be detected (*i.e*. containing CHX or CPC at one concentration lower than the MIC) were added to 10 mL BHI supplemented with the same concentration of antiseptic and incubated for 24 h at 37 °C. Afterwards, bacterial cultures were centrifuged and resulting pellets washed with fresh BHI medium to remove residual antiseptic solution. Pellets were subsequently resuspended in fresh BHI medium, bacterial solutions were adjusted to 10^7^ CFU/mL (OD_600_ ~ 0.5) and MIC determination was repeated as described before. This procedure was done for 10 consecutive passages.

A modified protocol was used for checking the increase in adaptation of *P. intermedia* and *P. gingivalis*, since their growth was fastidious and slow in the liquid cultures between the MIC determinations. Therefore, increasing the adaptation of *P. intermedia* and *P. gingivalis* was performed on agar plates containing a radial gradient of CHX or CPC created by an Autoplate® 4000 spiral plater (Spiral Biotech Inc., Norwood, USA). The antiseptic MIC value observed in the 96-well plate was used as the highest concentration in the gradient. Distribution of the antiseptics in an Archimedes spiral from the center to the edge established a ~1000-fold concentration gradient where the higher concentrations were located towards the center and the lower concentrations towards the edge. After 48 h of incubation, *P. intermedia* and *P. gingivalis* colonies situated in the area with the highest antiseptic concentration were taken, resuspended in BHI and adjusted to 10^7^ CFU/mL (OD_600_ ~ 0.5). MICs determination of *P. intermedia* and *P. gingivalis* were performed as described earlier for the other oral pathogens. Samples of the 10^th^ passage from all oral species were stored at −80 °C for further use. All experiments were repeated on three different days.

### Antiseptic and antibiotic cross-adaptation development

To examine cross-adaptation development to other antiseptics, a MIC analysis was performed for wild type (no previous exposure to CPC or CHX), CPC- and CHX-adapted species for CHX and CPC respectively, as described above. The MIC of CPC was determined for the wild type and CHX-adapted species and the MIC of CHX was determined for the wild type and CPC-adapted species.

In addition, cross-resistance development of CHX- and CPC-adapted species to four commonly used antibiotics (amoxicillin, azithromycin, metronidazole and tetracycline) was examined by means of ETEST® strips (BioMérieux, Brussels, Belgium). Overnight cultures of wild type, CHX- and CPC-adapted bacterial species were adjusted to 10^7^ CFU/mL (OD_600_ ~ 0.250) and inoculated on blood agar plates. ETEST® strips containing a concentration gradient of amoxicillin, azithromycin, metronidazole or tetracycline were placed on the blood agar plates and incubated under anaerobic conditions at 37 °C for 10 days. A calibrated (by means of a ruler) and standardized (fixed distance between photo camera and agar plate) photograph was taken from each agar plate. MIC values were determined visually as the antibiotic concentration at which the edge of the inhibition ellipse intersected with the sides of the antibiotic strip. When the MIC was lower than the lowest concentration present in the gradient, the value was estimated by extrapolation after measuring the distance (in mm) between the edge of the inhibition ellipse and the lowest concentration on the ETEST® strip using ImageJ (Imaging processing and Analysis in Java) software. All experiments were repeated on three different days.

### Reversibility of the (cross-)adaptation development

In order to verify if the induced adaptation and cross-adaptation development was reversible, adapted species were grown in absence of antiseptics on agar plates without antiseptics for 10 passages. Wild type controls were handled in the same way. Bacterial suspensions were subsequently adjusted to 10^7^ CFU/mL (OD_600_ ~ 0.5). MICs determinations were performed to re-evaluate the antiseptic adaptation and the antiseptic cross-adaptation, whereas ETEST® strips were used to re-determine the cross-adaptation to antibiotics. MIC and ETEST® experiments were performed as described previously. All experiments were repeated on three different days.

### Analysis of cell-surface hydrophobicity

Cell-surface hydrophobicity of wild type, CHX-adapted and CPC-adapted bacteria was measured by microbial adherence to n-hexadecane^53^. Bacterial suspensions were washed twice and re-suspended in phosphate-urea-magnesium sulfate buffer (100 mM sodium phosphate buffer, pH 7.1, 30 mM urea, 0.8 mM MgSO_4_) to an initial optical density at 550 nm (OD_550_) of 0.55–0.60. Four mL of bacterial suspension was mixed vigorously with 0.5 mL of n-hexadecane (Sigma-Aldrich, St. Louis, USA) for 30 s, and left at room temperature for 10 min. The OD_550_ of the lower aqueous phase was measured and hydrophobicity was represented as the percentage of adherence (% AD) to the hydrocarbon. The latter was calculated as: [% AD = (1 − A/I) × 100], where I equals the OD_550_ of the initial cell suspension and A equals the OD_550_ of the aqueous phase^54^. All experiments were repeated on three different days.

### Proteomic analysis

#### Protein purification

20 mL of overnight culture of the wild type, CHX-adapted and CPC-adapted strains was centrifuged (30 min at 4000 × *g*) and washed with physiological saline solution. Next, bacterial pellets were washed with 10 mL buffer A (50 mM HEPES, pH 7.5, 300 mM NaCl, 5 mM β-mercapto-ethanol, 1 mM EDTA) and centrifuged at 4 °C (30 min at 4000 × *g*). Supernatant was discarded and 20 mL of buffer B (buffer A supplemented with 1 µg/mL leupeptin, 0.1 mg/mL AEBSF (4-(2-aminoethyl)benzenesulfonyl fluoride hydrochloride)) was added to the bacterial pellet. Breaking of the cells was done by means of a Glen Creston Cell Homogenizer (pressure set to 20000–25000 psi) and suspensions were also sonicated (Branson digital sonifier 50/60 HZ) on ice during an alternating 2 min cycle (15 s pulses at 50% power, 30 s pauses on ice, until completion of 2 min of total sonication time)^55^.

#### Mass spectrometry experiments

For the mass spectrometry experiments, dithiothreitol (DTT) solution (final concentration 0.020 M) was added to the purified proteins and the mixture was incubated at room temperature for 15 min. Subsequently, iodoacetamide (IAA) was added to the solution (final concentration 0.050 M) followed by a 30-min incubation in the dark. Next, ammonium bicarbonate (ABC) (final concentration 0.11 M) was added to the samples (starting material 20 µg) together with trypsin (0.2 µg trypsin per 20 µg protein). Trypsin digestion was allowed to take place during at least 16 h at 37 °C to ensure complete proteolysis. Afterwards, resulting peptides were cleaned using C18 spin Columns (Thermo Fisher Scientific, Waltham, USA). Cleaned peptides were subsequently dried by Speedvac. Finally, the dried peptides were diluted in 10 µL 5% ammonium bicarbonate/acetonitrile (ACN) + 0.1% formic acid (FA) according to the manufacturer’s instructions for injection in the mass spectrometer (Q Exactive Orbitrap mass spectrometer, Thermo Fisher Scientific, Waltham, USA)^55^.

#### Analysis of mass spectrometry data

The peptide samples (5 µL of each sample) were digested and subsequently injected for UPLC separation by an Ultimate 3000 UPLC System (Dionex, Thermo Fisher Scientific, Waltham, USA), using an Acclaim PepMap100 pre-column (C18 3µm-100 Å, Thermo Fisher Scientific, Waltham, USA) and a C18 PepMap RSLC (2 μm, 50 μm-15 cm, Thermo Fisher Scientific, Waltham, USA) using a linear gradient (300 μL/min) of 0–4% buffer B (80% ACN, 0.08% FA) for 3 min, 4–10% B for 12 min, 10–35% for 20 min, 35–65% for 5 min, 65–95% for 1 min, 95% for 10 min, 95–5% for 1 min, and 5% for 10 min. The operational settings of the mass spectrometer and data acquisition procedure were identical to those described earlier by Khodaparast *et al*.^55^. With the purpose of identification, all raw data were converted into mgf.files using Proteome Discover version 1.4 (Thermo Fisher Scientific, Waltham, USA) and processed with MASCOT version 2.2.06 (Matrix Science Ltd, London, UK) against the Uniprot *A. actinomycetemcomitans*, *F. nucleatum*, *P. gingivalis* or *P. intermedia* database. The parameters used to search with MASCOT are described elsewhere^55^. MASCOT results were imported to Scaffold (version 3.6.3). The parameters used in Scaffold for protein identification were retaining proteins with 99% confidence and containing at least two identified peptides with a confidence level of 95%.

### Statistical analysis

For each component and species apart, a weighted linear mixed model with run as random factor and treatment and time as crossed fixed factors was fit. Weights were inversely proportional to the variance of each combination of the fixed factors. Normality of the residuals was assessed by means of a normal quantile plot. The comparison between treatments was performed between treatments for every time separately and a correction for simultaneous hypothesis testing according to Sidak was performed.

## Supplementary information

Supplementary Information

## Data Availability

The authors declare that all data supporting the findings of this study are available within the paper and its supplementary information files.
