# Supplementary material for: Development of antiseptic adaptation and cross-adapatation in selected oral pathogens in vitro
Source: Sci Rep. 2019 Jun 6;9:8326. doi: 10.1038/s41598-019-44822-y (PMC6554408; doi:10.1038/s41598-019-44822-y)
Supplement: Supplementary file 1 — Supplementary Information [file 41598_2019_44822_MOESM1_ESM.pdf]

SUPPLEMENTARY INFORMATION

Development of antiseptic adaptation and cross-adapatation in selected oral pathogens in vitro

Tim Verspecht[1#], Esteban Rodriguez Herrero[1#], Ladan Khodaparast[2,3], Laleh Khodaparast[2,3], Nico Boon[4], Kristel Bernaerts[5], Marc Quirynen[1,6], Wim Teughels[1,6]*

[#] Co-first authors

[1]Department of Oral Health Sciences, University of Leuven (KU Leuven), Kapucijnenvoer 33, 3000 Leuven, Belgium.

[2]Switch Laboratory, VIB Center for Brain and Disease Research, Herestraat 49, 3000 Leuven, Belgium.

[3] Switch Laboratory, Department of Cellular and Molecular Medicine, University of Leuven (KU Leuven), Herestraat 49, 3000, Leuven, Belgium.

[4]Center for Microbial Ecology and Technology (CMET), Ghent University (UGent), Coupure links 653, 9000 Gent, Belgium.

[5]Bio- and Chemical Systems Technology, Reactor Engineering and Safety, Department of Chemical Engineering, University of Leuven (KU Leuven), Leuven Chem&Tech, Celestijnenlaan 200F (bus 2424), 3001 Leuven, Belgium.

[6]Dentistry, University Hospitals Leuven, Kapucijnenvoer 33, 3000 Leuven, Belgium.

E-mail addresses authors: tim.verspecht@kuleuven.be, estebanrh89@hotmail.com, ladan.khodaparast@kuleuven.vib.be, laleh.khodaparast@kuleuven.vib.be, nico.boon@ugent.be, kristel.bernaerts@kuleuven.be, marc.quirynen@med.kuleuven.be, wim.teughels@med.kuleuven.be

*** Corresponding author:**

Wim Teughels, KU Leuven, Department of Oral Health Sciences, Kapucijnenvoer 33, 3000 Leuven, Belgium. Phone: +32 (0)16 332 505, fax: +32 (0)16 332 484. E-mail: wim.teughels@med.kuleuven.be

Supplementary Table S1 Evaluation of antiseptic adaptation and its reversibility by determination of MIC values of CHX and CPC against selected oral pathogens

	Average MIC ± SD (µg/mL)		
**CHX**	Control	Adaptation (10 P)[1]	Reversibility (10 P)[2]
A. actinomycetemcomitans	4.0 ± 0.0	**10.0 ± 0.8***	**9.0 ± 0.8***
F. nucleatum	3.7 ± 0.5	**9.3 ± 0.9***	**9.0 ± 0.0***
P. gingivalis	7.0 ± 0.8	**9.3 ± 0.4***	8.3 ± 0.5
P. intermedia	2.0 ± 0.0	**8.0 ± 0.8***	**4.7 ± 0.5* •**
S. mutans	1.6 ± 0.5	**3.0 ± 0.0***	**3.0 ± 0.0***
S. sobrinus	1.9 ± 0.9	**5.0 ± 0.8***	**6.0 ± 0.0***
**CPC**	Control	Adaptation (10 P)[1]	Reversibility (10 P)[2]
A. actinomycetemcomitans	8.0 ± 0.8	**13.7 ± 0.5***	**8.0 ± 0.0•**
F. nucleatum	4.0 ± 0.0	**7.0 ± 1.2***	**7.3 ± 1.2***
P. gingivalis	4.7 ± 0.5	**8.7 ± 0.5***	**11.3 ± 1.7* •**
P. intermedia	2.7 ± 0.5	**10.7 ± 1.2***	**7.3 ± 0.5***
S. mutans	1.7 ± 0.5	**5.0 ± 0.9***	3.0 ± 0.0
S. sobrinus	0.9 ± 0.05	**5.0 ± 0.8***	**4.3 ± 0.5***

[1]Bacteria exposed to CHX or CPC during 10 passages

[2]Bacteria exposed to CHX or CPC during 10 passages and regrown in absence of antiseptics during 10 passages

MIC: Minimum Inhibitory Concentration

Statistically significant (*P* < 0.05) higher MIC values compared to the wild type control are marked with ‘*’ and shown in bold. Statistically significant (*P* < 0.05) different MIC values when comparing ‘[2]’ to ‘[1]’ are marked with ‘•’ and shown in bold. N = 3.

Supplementary Table S2 Evaluation of antiseptic cross-adaptation and its reversibility by determination of MICs of CHX and CPC against selected oral pathogens

	Average MIC ± SD (µg/mL)		
**CHX**	Control	Adaptation (10 P)[1]	Reversibility (10 P)[2]
A. actinomycetemcomitans	4.0 ± 0.0	4.7 ± 0.5	**10.0 ± 0.8* •**
F. nucleatum	3.7 ± 0.5	2.7 ± 0.5	4.7 ± 0.9
P. gingivalis	7.0 ± 0.8	**9.0 ± 0.0***	8.7 ± 0.5
P. intermedia	2.0 ± 0.0	**7.7 ± 0.9***	**3.0 ± 0.8 •**
S. mutans	1.6 ± 0.5	1.0 ± 0.0	**3.0 ± 0.8 •**
S. sobrinus	1.9 ± 0.9	**4.0 ± 0.0***	**6.0 ± 0.0* •**
**CPC**	Control	Adaptation (10 P)[1]	Reversibility (10 P)[2]
A. actinomycetemcomitans	8.0 ± 0.8	8.3 ± 0.5	8.3 ± 0.5
F. nucleatum	4.0 ± 0.0	**6.7 ± 0.5***	**8.3 ± 0.5* •**
P. gingivalis	4.7 ± 0.5	**7.3 ± 0.5***	**7.7 ± 0.5***
P. intermedia	2.7 ± 0.5	**10.0 ± 0.5***	**9.0 ± 0.8***
S. mutans	1.7 ± 0.5	1.0 ± 0.0	**3.0 ± 0.0* •**
S. sobrinus	0.9 ± 0.05	**2.7 ± 0.5***	**4.0 ± 0.0* •**

[1]CPC- or CHX-adapted bacteria (10th passage) exposed to CHX or CPC, respectively, during 10 passages

[2]CPC- or CHX-adapted bacteria (10th passage) exposed to CHX or CPC during 10 passages and re-grown in absence of antiseptics during 10 passages.

MIC: Minimum Inhibitory Concentration

Statistically significant ($P < 0.05$) higher MIC values compared to the wild type control are marked with ‘*’ and shown in bold. Statistically significant ($P < 0.05$) different MIC values when comparing ‘[2]’ to ‘[1]’ are marked with ‘•’ and shown in bold. N = 3.

Supplementary Table S3 Evaluation of antibiotic cross-adaptation and its reversibility by MIC determination of four commonly used antibiotics

	Average MIC ± SD (µg/mL)					
	**Amoxicillin**			**Azithromycin**		
**CHX**	Control	Adaptation (10 P)[1]	Reversibility (10 P)[2]	Control	Adaptation (10 P)[1]	Reversibility (10 P)[2]
A. actinomycetemcomitans	0.563 ± 0.135	0.875 ± 0.102	0.875 ± 0.177	0.708 ± 0.212	0.917 ± 0.059	0.875 ± 0.000
F. nucleatum	0.016 ± 0.000	0.016 ± 0.000	**0.833 ± 0.059* •**	1.917 ± 0.118	1.750 ± 0.204	1.500 ± 0.353
P. gingivalis	0.004 ± 0.001	0.002 ± 0.000	**0.008 ± 0.000* •**	0.064 ± 0.000	**0.168 ± 0.031***	**0.074 ± 0.014•**
P. intermedia	0.016 ± 0.000	0.016 ± 0.000	0.011 ± 0.003	0.142 ± 0.035	0.179 ± 0.015	0.220 ± 0.024
S. mutans	0.032 ± 0.007	0.035 ± 0.003	**0.064 ± 0.000* •**	0.179 ± 0.015	0.230 ± 0.028	0.190 ± 0.000
S. sobrinus	0.018 ± 0.003	0.033 ± 0.010	**0.115 ± 0.015* •**	0.184 ± 0.098	**0.420 ± 0.028***	**0.500 ± 0.000***

	**Metronidazole**			**Tetracycline**		
**CHX**	Control	Adaptation (10 P)[1]	Reversibility (10 P)[2]	Control	Adaptation (10 P)[1]	Reversibility (10 P)[2]
A. actinomycetemcomitans	N.I.	N.I.	N.I.	0.200 ± 0.014	0.110 ± 0.000	0.125 ± 0.000
F. nucleatum	0.009 ± 0.002	0.017 ± 0.003	**0.037 ± 0.007* •**	0.022 ± 0.002	0.058 ± 0.026	**0.158 ± 0.026* •**
P. gingivalis	0.010 ± 0.002	0.005 ± 0.000	0.005 ± 0.000	0.034 ± 0.010	0.029 ± 0.002	0.028 ± 0.000
P. intermedia	0.080 ± 0.012	0.081 ± 0.000	**0.179 ± 0.015* •**	0.061 ± 0.013	0.047 ± 0.000	0.081 ± 0.030
S. mutans	N.I.	N.I.	N.I.	0.337 ± 0.061	0.230 ± 0.014	0.147 ± 0.015
S. sobrinus	N.I.	N.I.	N.I.	0.220 ± 0.024	**0.315 ± 0.000***	**0.178 ± 0.040•**

[1]Bacteria exposed to CHX during 10 passages
[2]Bacteria exposed to CHX during 10 passages and regrown in absence of antiseptics during 10 passages
MIC: Minimum Inhibitory Concentration
Statistically significant ($P < 0.05$) higher MIC values compared to the wild type control are marked with '*****' and shown in bold. Statistically significant ($P < 0.05$) different MIC values when comparing '[2]' to '[1]' are marked with '•' and shown in bold. N = 3. N.I. = not inhibited.

Supplementary Table S4 Evaluation of antibiotic cross-adaptation and its reversibility by MIC determination of four commonly used antibiotics

	**Average MIC ± SD (µg/mL)**					
	**Amoxicillin**			**Azithromycin**		
**CPC**	Control	Adaptation (10 P)[1]	Reversibility (10 P)[2]	Control	Adaptation (10 P)[1]	Reversibility (10 P)[2]
A. actinomycetemcomitans	0.563 ± 0.134	0.833 ± 0.118	**1.000 ± 0.000***	0.708 ± 0.212	1.208 ± 0.386	0.875 ± 0.000
F. nucleatum	0.016 ± 0.000	0.021 ± 0.002	**0.028 ± 0.004***	1.917 ± 0.118	1.167 ± 0.236	1.670 ± 0.118
P. gingivalis	0.004 ± 0.001	0.005 ± 0.003	0.005 ± 0.000	0.064 ± 0.000	**0.220 ± 0.024***	**0.875 ± 0.000*** •
P. intermedia	0.016 ± 0.000	0.024 ± 0.005	0.018 ± 0.003	0.142 ± 0.035	0.147 ± 0.031	0.160 ± 0.063
S. mutans	0.032 ± 0.007	0.037 ± 0.019	0.069 ± 0.007	0.179 ± 0.015	**0.380 ± 0.000***	**0.250 ± 0.000*** •
S. sobrinus	0.018 ± 0.003	0.026 ± 0.002	**0.110 ± 0.013*** •	0.184 ± 0.098	0.380 ± 0.031	**0.500 ± 0.000*** •

	**Metronidazole**			**Tetracycline**		
**CPC**	Control	Adaptation (10 P)[1]	Reversibility (10 P)[2]	Control	Adaptation (10 P)[1]	Reversibility (10 P)[2]
A. actinomycetemcomitans	N.I.	N.I.	N.I.	0.200 ± 0.014	0.157 ± 0.045	0.110 ± 0.000
F. nucleatum	0.009 ± 0.002	0.023 ± 0.007	0.017 ± 0.002	0.022 ± 0.002	0.029 ± 0.004	0.034 ± 0.008
P. gingivalis	0.010 ± 0.002	0.007 ± 0.000	0.004 ± 0.000	0.034 ± 0.010	0.037 ± 0.007	0.032 ± 0.006
P. intermedia	0.080 ± 0.012	0.200 ± 0.128	0.079 ± 0.012	0.061 ± 0.014	0.081 ± 0.018	0.069 ± 0.007
S. mutans	N.I.	N.I.	N.I.	0.337 ± 0.061	0.173 ± 0.047	0.158 ± 0.026
S. sobrinus	N.I.	N.I.	N.I.	0.220 ± 0.024	0.158 ± 0.045	0.230 ± 0.028

[1]Bacteria exposed to CPC during 10 passages
[2]Bacteria exposed to CPC during 10 passages and regrown in absence of antiseptics during 10 passages
MIC: Minimum Inhibitory Concentration
Statistically significant (P < 0.05) higher MIC values compared to the wild type control are marked with '*****' and shown in bold. Statistically significant (P < 0.05) different MIC values when comparing '[2]' to '[1]' are marked with '•' and shown in bold. N = 3. N.I. = not inhibited.

Supplementary Table S5 Overview of upregulated or uniquely present proteins in antiseptic-adapted *A. actinomyctemcomitans*

Adapted species	Protein name (accession number)	Protein function	
		Native	Associated with resistance/virulence
**CHX-adapted *Aa***	Transketolase ° (A0A142G0W1)	Participation in pentose-phosphate pathway	Cell wall biosynthesis [1]<br>Oxidative stress protection [2]<br>Drug resistance [3]
	Phosphate acetyltransferase ° (A0A142FZH6)	Taurine/hypotaurine metabolism, pyruvate metabolism and propanoate metabolism	Antibiotic and oxidative stress resistance [4]
	Aspartate ammonia-lyase ° (A0A142G0I3)	Fumarate production	Resistance to antibiotics/persistence [5,6]
	2,3-bisphosphoglycerate-dependent phosphoglycerate mutase ° (A0A142G1M9)	Essential glycolytic and gluconeogenic pathways	Upregulated in antibiotic-resistant bacteria [7]
	Triosephosphate isomerase ° (A0A142G0X5)	Important role in glycolysis	Upregulated in antibiotic-resistant bacteria [8]<br>Essential for infection [9]<br>Biosynthetic precursors for cell wall components and nucleic acids [10]
	Thiol:disulfide interchange protein ° (A0A142G1J9)	Rescue of secreted proteins damaged by oxidative stress	Oxidative stress protection [11]<br>Defects in this protein increase sensitivity to antibiotics [12]
	4-hydroxy-tetrahydrodipicolinate reductase ° (A0A142G2X6)	Lysine biosynthesis	Increase in peptidoglycan synthesis [13]<br>Found in bacterial species exposed to sub-lethal antibiotic concentrations [13]
	Phosphoserine aminotransferase (A0A142FYA8)	Amino acid metabolism	Upregulated in multi-drug resistant bacteria [14]
	Na(+)-translocating NADH-quinone reductase subunit A ° (A0A142FZX6)	Respiratory chain<br>Oxidation-reduction processes<br>Sodium ion transport	Alterations of these protein contribute to antibiotic resistance [15]<br>Involved in the physiology of pathogens [16]<br>Related to elevated efflux pump activity and decreased OM permeability [15]
	D-3-phosphoglycerate dehydrogenase ***** (A0A142FZ29)	Amino acid transport and metabolism	Upregulated in presence of cell wall-active antibiotics [17]<br>Bacterial adhesion and invasion [18]
	Tryptophan--tRNA ligase ****** (A0A142G174)	Tryptophan metabolism and aminoacyl-tRNA biosynthesis	Confers high-level resistance to antibiotics [19]
	Hydrogenase nickel incorporation protein HypB ***** (A0A142FXT5)	Incorporation of nickel	Significantly associated with resistance to several antibiotics [20]<br>Urease and hydrogenase activities in pathogenic bacteria [21]<br>Involved in colonization and resistance [21]
	C4-dicarboxylate ABC transporter ***** (A0A142G2C4)	Transmembrane transport	Highly upregulated in response to antibiotics [22]<br>Resistance to antibiotics [23]

**CPC-adapted *Aa***	Pyruvate kinase ° (A0A142G339)	Important role in glycolysis	Present in multi-resistant antibiotic species [24]<br>Essential in MRSA [25]
	2,3-bisphosphoglycerate-dependent phosphoglycerate mutase ° (A0A142G1M9)	Essential glycolytic and gluconeogenic pathways	Upregulated in antibiotic-resistant species [7]
	Probable cytosol aminopeptidase pepA ° (A0A142FXS3)	Hydrolysis of N-terminal amino acid residues	Alterations of the encoding gene are related to increased antibiotic resistance [26]<br>Involved in epithelial cell cytotoxicity *in vitro* and virulence [27]
	ABC transporter ATP-binding protein ° (A0A142FY59)	Transmembrane transport	Actively pumps the drug out of the bacterial cell [28]
	6-phosphogluconate dehydrogenase (decarboxylating) ° (A0A142FZE4)	Involved in pentose-phosphate pathway	Produced by MRSA and antibiotic-resistant species [29,30]<br>Lipopolysaccharide production [31]
	Protein translocase subunit SecD ° (A0A142FYD1)	Protein translocation	Involved in cell envelope formation [32]<br>Export of β-lactamases [33]
	4-hydroxy-tetrahydrodipicolinate reductase ° (A0A142G2X6)	Lysine biosynthesis	Increase of peptidoglycan synthesis [13]<br>Found in bacterial species exposed to sub-lethal antibiotic concentrations [13]
	Hydrogenase 3 large subunit ° (A0A142G2F2)	Hydrogen production	Neutralization of OH• (hydroxyl radicals) produced by bactericidal antibiotics and phagocytes [34]
	D-3-phosphoglycerate dehydrogenase * (A0A142FZ29)	Amino acid transport and metabolism	Upregulated in presence of cell wall-active antibiotics [17]<br>Bacterial adhesion and invasion [18]
	Hydrogenase nickel incorporation protein HypB * (A0A142FXT5)	Nickel incorporation	Significantly associated with resistance to several antibiotics [20]<br>Urease and hydrogenase activities in pathogenic bacteria [21]<br>Involved in colonization and resistance [21]
	C4-dicarboxylate ABC transporter ***** (A0A142G2C4)	Transmembrane transport	Highly upregulated in response to antibiotics [22]<br>Resistance to antibiotics [23]
	Ribulose-phosphate 3-epimerase ****** (A0A142G176)	Involved in pentose-phosphate pathway	Essential in arabinans production for cell wall biosynthesis [35]
	Nickel ABC transporter substrate-binding protein ****** (A0A142G082)	Transmembrane transport	Translocation of antibiotics in pathogens [36]<br>Resistance to antibiotics [36]
	ATP synthase subunit delta ****** (A0A142G0F4)	Transmembrane transport	High activity in multi-drug resistant bacteria [37]<br>Important influence on the efflux pumps [38]

CHX-adapted *Aa*: *A. actinomycetemcomitans* exposed to chlorhexidine during 10 passages; CPC-adapted *Aa*: *A. actinomycetemcomitans* exposed to cetylpyridinium chloride during 10 passages.

° Proteins also detected in wild type *A. actinomycetemcomitans* but significantly upregulated in the adapted species ($P < 0.05$).

* Proteins uniquely present in both CHX-adapted and CPC-adapted *A. actinomycetemcomitans* compared to the wild type species.

** Proteins uniquely present in either CHX- or CPC-adapted *A. actinomycetemcomitans* compared to the wild type species.

References used in Supplementary Table S5

1 Wolucka, B. A. Biosynthesis of D-arabinose in mycobacteria - a novel bacterial pathway with implications for antimycobacterial therapy. *FEBS J* **275**, 2691-2711, doi:10.1111/j.1742-4658.2008.06395.x (2008).

2 Kartal, B. & Palabiyik, B. Thiamine leads to oxidative stress resistance via regulation of the glucose metabolism. *Cell Mol Biol (Noisy-le-grand)* **65**, 73-77 (2019).

3 Domain, F., Bina, X. R. & Levy, S. B. Transketolase A, an enzyme in central metabolism, derepresses the marRAB multiple antibiotic resistance operon of Escherichia coli by interaction with MarR. *Mol Microbiol* **66**, 383-394, doi:10.1111/j.1365-2958.2007.05928.x (2007).

4 Li, X. *et al.* Tigecycline resistance in Acinetobacter baumannii mediated by frameshift mutation in plsC, encoding 1-acyl-sn-glycerol-3-phosphate acyltransferase. *Eur J Clin Microbiol Infect Dis* **34**, 625-631, doi:10.1007/s10096-014-2272-y (2015).

5 Gautam, A., Vinson, H. M., Gibbs, P. S., Olet, S. & Barigye, R. Proteomic analysis of multidrug resistant Escherichia coli strains from scouring calves. *Vet Microbiol* **151**, 363-371, doi:10.1016/j.vetmic.2011.03.032 (2011).

6 Bernier, S. P., Letoffe, S., Delepierre, M. & Ghigo, J. M. Biogenic ammonia modifies antibiotic resistance at a distance in physically separated bacteria. *Mol Microbiol* **81**, 705-716, doi:10.1111/j.1365-2958.2011.07724.x (2011).

7 Lin, X., Kang, L., Li, H. & Peng, X. Fluctuation of multiple metabolic pathways is required for Escherichia coli in response to chlortetracycline stress. *Mol Biosyst* **10**, 901-908, doi:10.1039/c3mb70522f (2014).

8 Mukherjee, S., Roychowdhury, A., Dutta, D. & Das, A. K. Crystal structures of triosephosphate isomerase from methicillin resistant Staphylococcus aureus MRSA252 provide structural insights into novel modes of ligand binding and unique conformations of catalytic loop. *Biochimie* **94**, 2532-2544, doi:10.1016/j.biochi.2012.07.001 (2012).

9 Trujillo, C. *et al.* Triosephosphate isomerase is dispensable in vitro yet essential for Mycobacterium tuberculosis to establish infection. *MBio* **5**, e00085, doi:10.1128/mBio.00085-14 (2014).

10 Henderson, B. & Martin, A. Bacterial virulence in the moonlight: multitasking bacterial moonlighting proteins are virulence determinants in infectious disease. *Infect Immun* **79**, 3476-3491, doi:10.1128/IAI.00179-11 (2011).

11 Dahl, J. U., Gray, M. J. & Jakob, U. Protein quality control under oxidative stress conditions. *J Mol Biol* **427**, 1549-1563, doi:10.1016/j.jmb.2015.02.014 (2015).

12 Bocian-Ostrzycka, K. M., Grzeszczuk, M. J., Banas, A. M. & Jagusztyn-Krynicka, E. K. Bacterial thiol oxidoreductases - from basic research to new antibacterial strategies. *Appl Microbiol Biotechnol* **101**, 3977-3989, doi:10.1007/s00253-017-8291-8 (2017).

13 Lee, C. R., Lee, J. H., Park, K. S., Jeong, B. C. & Lee, S. H. Quantitative proteomic view associated with resistance to clinically important antibiotics in Gram-positive bacteria: a systematic review. *Front Microbiol* **6**, 828, doi:10.3389/fmicb.2015.00828 (2015).

14 Piras, C. *et al.* Comparative proteomics to evaluate multi drug resistance in Escherichia coli. *Mol Biosyst* **8**, 1060-1067, doi:10.1039/c1mb05385j (2012).

15 Dibrov, P., Dibrov, E. & Pierce, G. N. Na+-NQR (Na+-translocating NADH:ubiquinone oxidoreductase) as a novel target for antibiotics. *FEMS Microbiol Rev* **41**, 653-671, doi:10.1093/femsre/fux032 (2017).

16 Muras, V., Dogaru-Kinn, P., Minato, Y., Hase, C. C. & Steuber, J. The Na+-Translocating NADH:Quinone Oxidoreductase Enhances Oxidative Stress in the Cytoplasm of Vibrio cholerae. *J Bacteriol* **198**, 2307-2317, doi:10.1128/JB.00342-16 (2016).

17 Utaida, S. *et al.* Genome-wide transcriptional profiling of the response of Staphylococcus aureus to cell-wall-active antibiotics reveals a cell-wall-stress stimulon. *Microbiology* **149**, 2719-2732, doi:10.1099/mic.0.26426-0 (2003).

18 Yasuda, M. *et al.* Pseudomonas aeruginosa serA Gene Is Required for Bacterial Translocation through Caco-2 Cell Monolayers. *PLoS One* **12**, e0169367, doi:10.1371/journal.pone.0169367 (2017).

19 Vecchione, J. J. & Sello, J. K. Regulation of an auxiliary, antibiotic-resistant tryptophanyl-tRNA synthetase gene via ribosome-mediated transcriptional attenuation. *J Bacteriol* **192**, 3565-3573, doi:10.1128/JB.00290-10 (2010).

20 Jones, K. R., Cha, J. H. & Merrell, D. S. Who's Winning the War? Molecular Mechanisms of Antibiotic Resistance in Helicobacter pylori. *Curr Drug ther* **3**, 190-203 (2008).

21 Xia, W., Li, H., Yang, X., Wong, K. B. & Sun, H. Metallo-GTPase HypB from Helicobacter pylori and its interaction with nickel chaperone protein HypA. *J Biol Chem* **287**, 6753-6763, doi:10.1074/jbc.M111.287581 (2012).

22 Lin, J. T., Connelly, M. B., Amolo, C., Otani, S. & Yaver, D. S. Global transcriptional response of Bacillus subtilis to treatment with subinhibitory concentrations of antibiotics that inhibit protein synthesis. *Antimicrob Agents Chemother* **49**, 1915-1926, doi:10.1128/AAC.49.5.1915-1926.2005 (2005).

23 Tang, F. & Saier, M. H., Jr. Transport proteins promoting Escherichia coli pathogenesis. *Microb Pathog* **71-72**, 41-55, doi:10.1016/j.micpath.2014.03.008 (2014).

24 Zoraghi, R. *et al.* Identification of pyruvate kinase in methicillin-resistant Staphylococcus aureus as a novel antimicrobial drug target. *Antimicrob Agents Chemother* **55**, 2042-2053, doi:10.1128/AAC.01250-10 (2011).

25 Zoraghi, R. *et al.* Functional analysis, overexpression, and kinetic characterization of pyruvate kinase from methicillin-resistant Staphylococcus aureus. *Biochemistry* **49**, 7733-7747, doi:10.1021/bi100780t (2010).
26 Suzuki, H., Kamatani, S., Kim, E. S. & Kumagai, H. Aminopeptidases A, B, and N and dipeptidase D are the four cysteinylglycinases of Escherichia coli K-12. *J Bacteriol* **183**, 1489-1490, doi:10.1128/JB.183.4.1489-1490.2001 (2001).
27 Hauser, A. R., Kang, P. J. & Engel, J. N. PepA, a secreted protein of Pseudomonas aeruginosa, is necessary for cytotoxicity and virulence. *Mol Microbiol* **27**, 807-818 (1998).
28 Zhou, J. *et al.* An important role of a "probable ATP-binding component of ABC transporter" during the process of Pseudomonas aeruginosa resistance to fluoroquinolone. *Proteomics* **6**, 2495-2503, doi:10.1002/pmic.200501354 (2006).
29 Lundberg, B. E., Wolf, R. E., Jr., Dinauer, M. C., Xu, Y. & Fang, F. C. Glucose 6-phosphate dehydrogenase is required for Salmonella typhimurium virulence and resistance to reactive oxygen and nitrogen intermediates. *Infect Immun* **67**, 436-438 (1999).
30 Musser, J. M. & Kapur, V. Clonal analysis of methicillin-resistant Staphylococcus aureus strains from intercontinental sources: association of the mec gene with divergent phylogenetic lineages implies dissemination by horizontal transfer and recombination. *J Clin Microbiol* **30**, 2058-2063 (1992).
31 Burrows, L. L., Charter, D. F. & Lam, J. S. Molecular characterization of the Pseudomonas aeruginosa serotype O5 (PAO1) B-band lipopolysaccharide gene cluster. *Mol Microbiol* **22**, 481-495, doi:10.1046/j.1365-2958.1996.1351503.x (1996).
32 Kostakioti, M., Newman, C. L., Thanassi, D. G. & Stathopoulos, C. Mechanisms of protein export across the bacterial outer membrane. *J Bacteriol* **187**, 4306-4314, doi:10.1128/JB.187.13.4306-4314.2005 (2005).
33 Pradel, N., Delmas, J., Wu, L. F., Santini, C. L. & Bonnet, R. Sec- and Tat-dependent translocation of beta-lactamases across the Escherichia coli inner membrane. *Antimicrob Agents Chemother* **53**, 242-248, doi:10.1128/AAC.00642-08 (2009).
34 Nie, W. *et al.* Hydrogenase: the next antibiotic target? *Clin Sci (Lond)* **122**, 575-580, doi:10.1042/CS20110396 (2012).
35 Akana, J. *et al.* D-Ribulose 5-phosphate 3-epimerase: functional and structural relationships to members of the ribulose-phosphate binding (beta/alpha)8-barrel superfamily. *Biochemistry* **45**, 2493-2503, doi:10.1021/bi052474m (2006).
36 Tanaka, K. J., Song, S., Mason, K. & Pinkett, H. W. Selective substrate uptake: The role of ATP-binding cassette (ABC) importers in pathogenesis. *Biochim Biophys Acta Biomembr* **1860**, 868-877, doi:10.1016/j.bbamem.2017.08.011 (2018).
37 Ahmad, Z., Okafor, F., Azim, S. & Laughlin, T. F. ATP synthase: a molecular therapeutic drug target for antimicrobial and antitumor peptides. *Curr Med Chem* **20**, 1956-1973 (2013).
38 Balemans, W. *et al.* Novel antibiotics targeting respiratory ATP synthesis in Gram-positive pathogenic bacteria. *Antimicrob Agents Chemother* **56**, 4131-4139, doi:10.1128/AAC.00273-12 (2012).

Supplementary Table S6 Overview of upregulated or uniquely present proteins in antiseptic-adapted *F. nucleatum*

Adapted species	Protein name (accession number)	Protein function	
		Native	Associated to resistance/virulence
**CHX-adapted *Fn***	Possible bacterioferrin ˚ (A5TUE5)	Iron acquisition	Absence of iron reduces resistance to antibiotics [1]<br>Protects against antibiotics [1]<br>Upregulated in antibiotic-resistant bacteria [2]
	Peroxiredoxin ˚ (A5TS03)	Antioxidant effect	Increases its activity in presence of antibiotics [3]<br>Upregulated in presence of oxidants and persistence *in vivo* [4]<br>Influences bacterial virulence [5]
	Beta-lysine 5,6-aminomutase ˚ (A5TV47)	Participation in lysine degradation	Protects against oxidative stress [6]<br>Acetate produced by lysine fermentation increases tolerance to antibiotics [6,7]
	Acyl-CoA dehydrogenase ˚ (A5TY59)	Fatty acid β-oxidation	Cell wall and membranes biosynthesis [8]<br>Active in stress response [9]<br>Involved in bacterial survival and virulence [10]
	Copper (Cu2+)-exporting ATPase ***** (A5TT66)	Exports copper through the membrane	Increases the amount of positive charges, thereby reducing influx and adhesion of antiseptics to bacteria [11,12]
	Possible cobalamin/iron (Fe3+)-siderophore ABC superfamily ATP-binding cassette transporter binding protein ***** (A5TTC9)	Transport activity	Related to the transport of peptide antibiotics, heme, drugs and siderophores [13]
	L-serine ammonia-lyase ***** (A5TUB6)	Amino acid transport and metabolism	Upregulated in response to DNA-damaging agents [14]
	Uncharacterized protein ***** (A5TY36)	Not identified	-
	Uncharacterized protein ****** (5TVL7)	Possible ABC transport	Possible relation with drug transport [13]
**CPC-adapted *Fn***	Alcohol dehydrogenase ˚ (A5TRM7)	Bacterial fermentation	Upregulated upon exposure to different environmental stresses [15]
	Tyrosine phenol-lyase ˚ (A5TS08)	Tyrosine and nitrogen metabolism	-
	Peroxiredoxin ˚ (A5TS03)	Antioxidant effect	Increases its activity in presence of antibiotics [3]<br>Upregulated in presence of oxidants and persistence *in vivo* [4]<br>Influences bacterial virulence [5]
	Beta-lysine 5,6-aminomutase ˚ (A5TV47)	Participation in lysine degradation	Protects against oxidative stress [6]<br>Acetate produced by lysine fermentation increases tolerance to antibiotics [6,7]
	Acyl-CoA dehydrogenase ˚ (A5TY59)	Fatty acid β-oxidation	Cell wall and membranes biosynthesis [8]<br>Active in stress response [9]<br>Involved in bacterial survival and virulence [10]
	Leucine--tRNA ligase ˚ (A5TRK3)	Amino acid and aminoacyl-tRNA biosynthesis	Associated with resistance to several antibiotics [16]
	Transketolase ˚ (A5TTB7)	Participation in the pentose-phosphate pathway	Cell wall biosynthesis [17]<br>Oxidative stress protection [18]

			Drug resistance [19]
	Flavodoxin ˚ (A5TXC7)	Modifies the redox potentials	Decreased efficacy of antibiotics via the reduction of antibiotics [20]
	Uncharacterized protein ***** (A5TY36)	Not identified	-
	Copper (Cu2+)-exporting ATPase ***** (A5TT66)	Exports copper through the membrane	Increases the amount of positive charges, thereby reducing influx and adhesion of antiseptics to bacteria [11,12]
	Possible cobalamin/iron (Fe3+)-siderophore ABC superfamily ATP binding cassette transporter binding protein ***** (A5TTC9)	Transport activity	Related to the transport of peptide antibiotics, heme, drugs and siderophores [13]
	L-serine ammonia-lyase ***** (A5TUB6)	Amino acid transport and metabolism	Upregulated in response to DNA-damaging agents [14]
	Uncharacterized protein ****** (A5TVD9)	Possible lipoprotein	-
	Uncharacterized protein ****** (A5TSH6)	Possible outer membrane protein	Possible influence in permeability and antibiotic resistance [21]

CHX-adapted *Fn*: *F. nucleatum* exposed to chlorhexidine during 10 passages; CPC-adapted *Fn*: *F. nucleatum* exposed to cetylpyridinium chloride during 10 passages.

˚ Proteins also detected in wild type *F. nucleatum* but significantly upregulated in the adapted species (*P* < 0.05).

* Proteins uniquely present in both CHX-adapted and CPC-adapted *F. nucleatum* compared to the wild type species.

** Proteins uniquely present in either CHX- or CPC-adapted *F. nucleatum* compared to the wild type species.

References used in Supplementary Table S6

1 Mehi, O. *et al.* Perturbation of iron homeostasis promotes the evolution of antibiotic resistance. *Mol Biol Evol* **31**, 2793-2804, doi:10.1093/molbev/msu223 (2014).

2 Kumar, B. *et al.* Proteomic analysis of Mycobacterium tuberculosis isolates resistant to kanamycin and amikacin. *J Proteomics* **94**, 68-77, doi:10.1016/j.jprot.2013.08.025 (2013).

3 Perkins, A., Nelson, K. J., Parsonage, D., Poole, L. B. & Karplus, P. A. Peroxiredoxins: guardians against oxidative stress and modulators of peroxide signaling. *Trends Biochem Sci* **40**, 435-445, doi:10.1016/j.tibs.2015.05.001 (2015).

4 Lu, J. & Holmgren, A. The thioredoxin antioxidant system. *Free Radic Biol Med* **66**, 75-87, doi:10.1016/j.freeradbiomed.2013.07.036 (2014).

5 Kaihami, G. H. *et al.* Involvement of a 1-Cys peroxiredoxin in bacterial virulence. *PLoS Pathog* **10**, e1004442, doi:10.1371/journal.ppat.1004442 (2014).

6 Berkovitch, F. *et al.* A locking mechanism preventing radical damage in the absence of substrate, as revealed by the x-ray structure of lysine 5,6-aminomutase. *Proc Natl Acad Sci U S A* **101**, 15870-15875, doi:10.1073/pnas.0407074101 (2004).

7 Zampieri, M. *et al.* Metabolic constraints on the evolution of antibiotic resistance. *Mol Syst Biol* **13**, 917, doi:10.15252/msb.20167028 (2017).

8 Parsons, J. B. & Rock, C. O. Bacterial lipids: metabolism and membrane homeostasis. *Prog Lipid Res* **52**, 249-276, doi:10.1016/j.plipres.2013.02.002 (2013).

9 Spector, M. P. *et al.* The medium-/long-chain fatty acyl-CoA dehydrogenase (fadF) gene of Salmonella typhimurium is a phase 1 starvation-stress response (SSR) locus. *Microbiology* **145 (Pt 1)**, 15-31, doi:10.1099/13500872-145-1-15 (1999).

10 Kang, Y., Zarzycki-Siek, J., Walton, C. B., Norris, M. H. & Hoang, T. T. Multiple FadD acyl-CoA synthetases contribute to differential fatty acid degradation and virulence in Pseudomonas aeruginosa. *PLoS One* **5**, e13557, doi:10.1371/journal.pone.0013557 (2010).

11 Bondarczuk, K. & Piotrowska-Seget, Z. Molecular basis of active copper resistance mechanisms in Gram-negative bacteria. *Cell Biol Toxicol* **29**, 397-405, doi:10.1007/s10565-013-9262-1 (2013).

12 McDonnell, G. & Russell, A. D. Antiseptics and disinfectants: activity, action, and resistance. *Clin Microbiol Rev* **12**, 147-179 (1999).

13 Davidson, A. L., Dassa, E., Orelle, C. & Chen, J. Structure, function, and evolution of bacterial ATP-binding cassette systems. *Microbiol Mol Biol Rev* **72**, 317-364, table of contents, doi:10.1128/MMBR.00031-07 (2008).

14 Newman, E. B., Ahmad, D. & Walker, C. L-Serine deaminase activity is induced by exposure of Escherichia coli K-12 to DNA-damaging agents. *J Bacteriol* **152**, 702-705 (1982).

15 Vidal, R., Lopez-Maury, L., Guerrero, M. G. & Florencio, F. J. Characterization of an alcohol dehydrogenase from the Cyanobacterium Synechocystis sp. strain PCC 6803 that responds to environmental stress conditions via the Hik34-Rre1 two-component system. *J Bacteriol* **191**, 4383-4391, doi:10.1128/JB.00183-09 (2009).

16 Francklyn, C. S. & Mullen, P. Progress and challenges in aminoacyl-tRNA synthetase-based therapeutics. *J Biol Chem* **294**, 5365-5385, doi:10.1074/jbc.REV118.002956 (2019).

17 Wolucka, B. A. Biosynthesis of D-arabinose in mycobacteria - a novel bacterial pathway with implications for antimycobacterial therapy. *FEBS J* **275**, 2691-2711, doi:10.1111/j.1742-4658.2008.06395.x (2008).

18 Kartal, B. & Palabiyik, B. Thiamine leads to oxidative stress resistance via regulation of the glucose metabolism. *Cell Mol Biol (Noisy-le-grand)* **65**, 73-77 (2019).

19 Domain, F., Bina, X. R. & Levy, S. B. Transketolase A, an enzyme in central metabolism, derepresses the marRAB multiple antibiotic resistance operon of Escherichia coli by interaction with MarR. *Mol Microbiol* **66**, 383-394, doi:10.1111/j.1365-2958.2007.05928.x (2007).

20 Francesco, V. D. *et al.* Mechanisms of Helicobacter pylori antibiotic resistance: An updated appraisal. *World J Gastrointest Pathophysiol* **2**, 35-41, doi:10.4291/wjgp.v2.i3.35 (2011).

21 Ghai, I. & Ghai, S. Understanding antibiotic resistance via outer membrane permeability. *Infect Drug Resist* **11**, 523-530, doi:10.2147/IDR.S156995 (2018).

Supplementary Table S7 Overview of upregulated or uniquely present proteins in antiseptic-adapted *P. gingivalis*

Adapted species	Protein name (accession number)	Protein function	
		Native	Associated with resistance/virulence
**CHX-adapted *Pg***	Type I glyceraldehyde-3-phosphate dehydrogenase ˚ (A0A254MTR4)	Participation in glycolysis	Decreased susceptibility to antibiotics [1]<br>Immune system evasion [2]<br>Bacterial colonization [3]
	Succinate-semialdehyde dehydrogenase ˚ (A0A134DQ97)	Glutamate and butyrate metabolism	Its upregulation increases antibiotic resistance [4]<br>Energy support of the efflux pumps [4]
	Triosephosphate isomerase ˚ (A0A1R4ADH4)	Important role in glycolysis	Upregulated in antibiotic-resistant bacteria [5]<br>Essential for infection [6]<br>Biosynthetic precursors for cell wall components and nucleic acids [5]
	Lys-gingipain 381 ˚ (P72194)	Cysteine proteinase activity	Hemolysis and iron uptake [7]<br>Degradation of host tissues [7,8]<br>Disrupts the functions of PMN leukocytes [7]<br>Bacterial co-aggregation [9]
	Hemagglutinin A ˚ (Q51845)	Hemagglutination	Hemolysis and iron uptake [10]<br>Cell colonization and invasion [10,11]<br>Immunogenic factor [11]
	DNA-directed RNA polymerase subunit alpha ˚ (A0A134DRA5)	RNA synthesis	Mutations of the encoding gene increase bacterial antibiotic and biocide resistance [12,13]
	Thiazole biosynthesis protein ˚ (A0A1R4DY74)	Thiamine pyrophosphate (vitamin B1)	Expressed in antibiotic-resistant species [14]<br>Resistance to nitric acid produced by neutrophils [15]
	Electron transfer flavoprotein subunit beta ˚ (A0A1R4AHJ9)	Fatty acid oxidation and amino acid metabolism	Upregulated after exposure to antibiotics [16]
	Cysteine--tRNA ligase ˚ (A0A254MUG4)	Cysteine metabolism and aminoacyl-tRNA biosynthesis	Significantly associated with resistance to several antibiotics [17]
	Galactokinase ˚ (A0A134DPY4)	Galactose catabolism	Expressed in antibiotic-resistant species [18]
	3-oxoacyl-ACP synthase ˚ (A0A254N3Q0)	Fatty acids biosynthesis	Upregulated in antibiotic-resistant species [19]
	RND transporter MFP subunit ˚ (A0A1R4AD39)	Transmembrane transport	Active efflux of many antibiotics and chemotherapeutical agents [20]
	Two-component system response regulator ˚ (A0A1R4AG47)	Detection and response to environmental changes	In response to extracellular glycopeptide antibiotics [21]<br>Regulation of many antibiotic resistance determinants and efflux pumps [21]
	Arginine-specific cysteine protease RgpA ***** (A0A1R4ACS4)	Protease activity	Host tissue degradation [7]<br>Resistance to host defence mechanisms [7,22]<br>Cell invasion [7,22]<br>Hemagglutination and iron uptake [7,22]

	Indolepyruvate oxidoreductase ***** (A0A134DQ80)	Oxidation-reduction process	Confers resistance to antibiotics [23]
	Chromosome partitioning protein ***** (A0A134DQY1)	Chromosome segregation during cell division or transmission of plasmids	Essential role in cell viability [24]<br>Transmission of plasmids conferring resistance to antibiotics [24]
	Elongation factor 4 ***** (A0A1R4AF73)	Translocase activity	Required for accurate and efficient protein synthesis under certain stress conditions [25]
	T9SS C-terminal target domain-containing protein ****** (A0A1R4AGQ7)	Transmembrane transport	Involved in the secretion of virulence factors (gingipains) [26,27]<br>Cell wall biosynthesis [26]<br>Heme acquisition [27]
	Lysine-specific cysteine protease Kgp ****** (A0A1R4DPT4)	Protease activity	Host tissue degradation [7]<br>Resistance to host defence mechanisms [7,22]<br>Cell invasion [7,22]<br>Hemagglutination and iron uptake [7,22]
	DUF5063 domain-containing protein ****** (A0A254MT42)	Unknown function	-
	Penicillin-binding protein 1A ****** (A0A1R4AH86)	Peptidoglycan synthesis	Cell wall synthesis, cell division and maintaining cellular structure [28]<br>For instance found in MRSA [29]
**CPC-adapted *Pg***	Glutamate dehydrogenase ° (A0A254MUS7)	Urea synthesis	Contributes to resistance to cell wall-active antibiotics [30]
	3-methyl-2-oxobutanoate dehydrogenase subunit VorB ° (A0A254MWY1)	Amino acid degradation<br>Synthesis of lipoic acid	Influences on membrane properties (*i.e.* fluidity)[31]<br>Antioxidant [32,33]
	Phosphoserine aminotransferase ° (A0A254N4I0)	Amino acid metabolism	Upregulated in multi-drug resistant bacteria [34]
	2-oxoglutarate oxidoreductase ° (A0A1R4DQ72)	Involved in Krebs cycle	Role in resistance to antibiotics [35]
	Hemagglutinin A ° (Q51845)	Hemagglutination	Hemolysis and iron uptake [10]<br>Cell colonization and invasion [10,11]<br>Immunogenic factor [11]
	DNA-directed RNA polymerase subunit alpha ° (A0A134DRA5)	RNA synthesis	Mutations of the encoding gene increase bacterial antibiotic and biocide resistance [12,13]
	UDP-glucose 4-epimerase ° (A0A1R4AFM2)	Galactose metabolism and glycoprotein or glycolipid synthesis	Absence of the encoding gene reduces bacterial antibiotic resistance [36]<br>Participation in LPS synthesis [36]
	Acyl-CoA dehydrogenase ° (A0A1R4DX65)	Fatty acid β-oxidation	Cell wall and membranes biosynthesis [37]<br>Active in stress response [38]<br>Involved in bacterial survival and virulence [37,38]
	67kDa fimbrillin ° (O83017)	Structural protein of the fimbriae	Cell adherence and invasion [39,40]<br>Induction of immune response [39,40]<br>Biofilm formation [39,40]

	Type I deoxyribonuclease HsdR ° (A0A134DPM0)	Endonuclease activity	Bacterial protection [41]
	Hemolysin secretion protein D ° (A0A1R4AFK1)	Membrane transport	Secretion of hemolytic toxins [42]<br>Involved in the structure of efflux channels [43]
	Ribose-phosphate pyrophosphokinase ° (A0A1R4DTK5)	Synthesis of nucleotides (purines and pyrimidines)	Detected in multi-resistant species [44]
	Electron transfer flavoprotein subunit beta ° (A0A1R4AHJ9)	Fatty acid oxidation and amino acid metabolism	Upregulated after exposure to antibiotics [16]
	Cysteine--tRNA ligase ° (A0A254MUG4)	Cysteine metabolism and aminoacyl-tRNA biosynthesis	Significantly associated with resistance to several antibiotics [17]
	Orotate phosphoribosyltransferase ° (A0A134DP74)	Pyrimidine biosynthesis	Essential bacterial gene [45]
	S-adenosylmethionine synthase ° (A0A1R4DRF4)	S-adenosyl-L-methionine biosynthesis and amino acid biosynthesis	Methylation reactions of DNA, RNA and proteins [46]<br>Quorum sensing molecules that trigger virulence in Gram-negatives [46]
	Ribosome-recycling factor ° (A0A134DMY5)	Ribosome recycling after completion of protein synthesis	Enhanced protein synthesis [47]
	Lon protease ° (A0A134DQ49)	Proteolytic activity	Increased by the presence of antibiotics [48]<br>Role in cell division, biofilm formation, capsule synthesis, haemolytic activity and motility [48]
	Two-component system response regulator ° (A0A1R4AG47)	Detection and response to environmental changes	In response to extracellular glycopeptide antibiotics [21]<br>Regulation of many antibiotic resistance determinants and efflux pumps [21]
	Patatin ° (A0A1R4AEM3)	Bacterial lipolytic enzyme	Associated with secretion systems which are related to virulence factors secretion and efflux of antibiotics [49,50]
	Succinate-semialdehyde dehydrogenase ° (A0A134DQ97)	Glutamate and butyrate metabolism	Its upregulation increases antibiotic resistance [4]<br>Energy support of the efflux pumps [4]
	Arginine-specific cysteine protease RgpA ***** (A0A1R4ACS4)	Protease activity	Host tissue degradation [7]<br>Resistance to host defence mechanisms [7,22]<br>Cell invasion [7,22]<br>Hemagglutination and iron uptake [7,22]
	Indolepyruvate oxidoreductase ***** (A0A134DQ80)	Oxidation-reduction process	Confers resistance to antibiotics [23]
	Chromosome partitioning protein ***** (A0A134DQY1)	Chromosome segregation during cell division or transmission of plasmids	Essential role in cell viability [24]<br>Transmission of plasmids conferring resistance to antibiotics [24]
	Elongation factor 4 ***** (A0A1R4AF73)	Translocase activity	Required for accurate and efficient protein synthesis under certain stress conditions [25]

	Phosphoglucosamine mutase ** (A0A142G2L6)	Oxidation-reduction process	Confers resistance to antibiotics [51]
	4-hydroxythreonine-4-phosphate dehydrogenase ** (A0A1R4AH54)	Vitamin B6 metabolism<br>Involved in cell synthesis	Vitamin B6 is required for motility/virulence in some bacteria [52]<br>Too low B6 increases susceptibility to antibiotics[53]
	tRNA(Glu)-specific nuclease WapA ** (A0A1R4DVU2)	Cell wall-associated protein<br>RNase WapA<br>Toxin WapA	Inhibits other bacteria, thereby increasing survival rates with respect to other bacteria [54]<br>Virulence activity [54]
	Uridylate kinase ** (A0A134DMY9)	Participation in pyrimidine metabolism	Expressed during the *in vivo* infection process [55]

CHX-adapted *Pg*: *P. gingivalis* exposed to chlorhexidine during 10 passages; CPC-adapted *Pg*: *P. gingivalis* exposed to cetylpyridinium chloride during 10 passages.

° Proteins also detected in wild type *P. gingivalis* but significantly upregulated in the adapted species ($P < 0.05$).

* Proteins uniquely present in both CHX-adapted and CPC-adapted *P. gingivalis* compared to the wild type species.

** Proteins uniquely present in either CHX- or CPC-adapted *P. gingivalis* compared to the wild type species.

References used in Supplementary Table S7

1 Fajardo, A. et al. The neglected intrinsic resistome of bacterial pathogens. PLoS One **3**, e1619, doi:10.1371/journal.pone.0001619 (2008).

2 Terao, Y., Yamaguchi, M., Hamada, S. & Kawabata, S. Multifunctional glyceraldehyde-3-phosphate dehydrogenase of Streptococcus pyogenes is essential for evasion from neutrophils. J Biol Chem **281**, 14215-14223, doi:10.1074/jbc.M513408200 (2006).

3 Kinoshita, H. et al. Cell surface Lactobacillus plantarum LA 318 glyceraldehyde-3-phosphate dehydrogenase (GAPDH) adheres to human colonic mucin. J Appl Microbiol **104**, 1667-1674, doi:10.1111/j.1365-2672.2007.03679.x (2008).

4 Su, H. C. et al. The development of ciprofloxacin resistance in Pseudomonas aeruginosa involves multiple response stages and multiple proteins. Antimicrob Agents Chemother **54**, 4626-4635, doi:10.1128/AAC.00762-10 (2010).

5 Mukherjee, S., Roychowdhury, A., Dutta, D. & Das, A. K. Crystal structures of triosephosphate isomerase from methicillin resistant Staphylococcus aureus MRSA252 provide structural insights into novel modes of ligand binding and unique conformations of catalytic loop. Biochimie **94**, 2532-2544, doi:10.1016/j.biochi.2012.07.001 (2012).

6 Trujillo, C. et al. Triosephosphate isomerase is dispensable in vitro yet essential for Mycobacterium tuberculosis to establish infection. MBio **5**, e00085, doi:10.1128/mBio.00085-14 (2014).

7 Li, N. & Collyer, C. A. Gingipains from Porphyromonas gingivalis - Complex domain structures confer diverse functions. Eur J Microbiol Immunol (Bp) **1**, 41-58, doi:10.1556/EuJMI.1.2011.1.7 (2011).

8 Sroka, A., Sztukowska, M., Potempa, J., Travis, J. & Genco, C. A. Degradation of host heme proteins by lysine- and arginine-specific cysteine proteinases (gingipains) of Porphyromonas gingivalis. J Bacteriol **183**, 5609-5616, doi:10.1128/JB.183.19.5609-5616.2001 (2001).

9 Kamaguchi, A. et al. Adhesins encoded by the gingipain genes of Porphyromonas gingivalis are responsible for co-aggregation with Prevotella intermedia. Microbiology **149**, 1257-1264, doi:10.1099/mic.0.25997-0 (2003).

10 Lamont, R. J. & Jenkinson, H. F. Life below the gum line: pathogenic mechanisms of Porphyromonas gingivalis. Microbiol Mol Biol Rev **62**, 1244-1263 (1998).

11 Kozarov, E. et al. Expression and immunogenicity of hemagglutinin A from Porphyromonas gingivalis in an avirulent Salmonella enterica serovar typhimurium vaccine strain. Infect Immun **68**, 732-739 (2000).

12 Condell, O. et al. Comparative analysis of Salmonella susceptibility and tolerance to the biocide chlorhexidine identifies a complex cellular defense network. Front Microbiol **5**, 373, doi:10.3389/fmicb.2014.00373 (2014).

13 Webber, M. A. et al. Parallel evolutionary pathways to antibiotic resistance selected by biocide exposure. J Antimicrob Chemother **70**, 2241-2248, doi:10.1093/jac/dkv109 (2015).

14 Drebes, J. et al. Structure of ThiM from Vitamin B1 biosynthetic pathway of Staphylococcus aureus - Insights into a novel pro-drug approach addressing MRSA infections. Sci Rep **6**, 22871, doi:10.1038/srep22871 (2016).

15 Wolak, N., Kowalska, E., Kozik, A. & Rapala-Kozik, M. Thiamine increases the resistance of baker's yeast Saccharomyces cerevisiae against oxidative, osmotic and thermal stress, through mechanisms partly independent of thiamine diphosphate-bound enzymes. FEMS Yeast Res **14**, 1249-1262, doi:10.1111/1567-1364.12218 (2014).

16 Stietz, M. S., Lopez, C., Osifo, O., Tolmasky, M. E. & Cardona, S. T. Evaluation of the electron transfer flavoprotein as an antibacterial target in Burkholderia cenocepacia. Can J Microbiol **63**, 857-863, doi:10.1139/cjm-2017-0350 (2017).

17 Francklyn, C. S. & Mullen, P. Progress and challenges in aminoacyl-tRNA synthetase-based therapeutics. J Biol Chem **294**, 5365-5385, doi:10.1074/jbc.REV118.002956 (2019).

18 Lee, C. R., Lee, J. H., Park, K. S., Jeong, B. C. & Lee, S. H. Quantitative proteomic view associated with resistance to clinically important antibiotics in Gram-positive bacteria: a systematic review. Front Microbiol **6**, 828, doi:10.3389/fmicb.2015.00828 (2015).

19 Taguchi, F. et al. A homologue of the 3-oxoacyl-(acyl carrier protein) synthase III gene located in the glycosylation island of Pseudomonas syringae pv. tabaci regulates virulence factors via N-acyl homoserine lactone and fatty acid synthesis. J Bacteriol **188**, 8376-8384, doi:10.1128/JB.00763-06 (2006).

20 Nikaido, H. & Takatsuka, Y. Mechanisms of RND multidrug efflux pumps. Biochim Biophys Acta **1794**, 769-781, doi:10.1016/j.bbapap.2008.10.004 (2009).

21 Tiwari, S. et al. Two-Component Signal Transduction Systems of Pathogenic Bacteria As Targets for Antimicrobial Therapy: An Overview. Front Microbiol **8**, 1878, doi:10.3389/fmicb.2017.01878 (2017).

22 O'Brien-Simpson, N. M., Pathirana, R. D., Walker, G. D. & Reynolds, E. C. Porphyromonas gingivalis RgpA-Kgp proteinase-adhesin complexes penetrate gingival tissue and induce proinflammatory cytokines or apoptosis in a concentration-dependent manner. Infect Immun **77**, 1246-1261, doi:10.1128/IAI.01038-08 (2009).

23 Brown, D. M., Upcroft, J. A., Dodd, H. N., Chen, N. & Upcroft, P. Alternative 2-keto acid oxidoreductase activities in Trichomonas vaginalis. Mol Biochem Parasitol **98**, 203-214 (1999).

24 Attaiech, L., Minnen, A., Kjos, M., Gruber, S. & Veening, J. W. The ParB-parS Chromosome Segregation System Modulates Competence Development in Streptococcus pneumoniae. MBio **6**, e00662, doi:10.1128/mBio.00662-15 (2015).
25 Qin, Y. et al. The highly conserved LepA is a ribosomal elongation factor that back-translocates the ribosome. Cell **127**, 721-733, doi:10.1016/j.cell.2006.09.037 (2006).
26 Gorasia, D. G. et al. Porphyromonas gingivalis Type IX Secretion Substrates Are Cleaved and Modified by a Sortase-Like Mechanism. PLoS Pathog **11**, e1005152, doi:10.1371/journal.ppat.1005152 (2015).
27 Lasica, A. M., Ksiazek, M., Madej, M. & Potempa, J. The Type IX Secretion System (T9SS): Highlights and Recent Insights into Its Structure and Function. Front Cell Infect Microbiol **7**, 215, doi:10.3389/fcimb.2017.00215 (2017).
28 Macheboeuf, P., Contreras-Martel, C., Job, V., Dideberg, O. & Dessen, A. Penicillin binding proteins: key players in bacterial cell cycle and drug resistance processes. FEMS Microbiol Rev **30**, 673-691, doi:10.1111/j.1574-6976.2006.00024.x (2006).
29 Berti, A. D. et al. Penicillin Binding Protein 1 Is Important in the Compensatory Response of Staphylococcus aureus to Daptomycin-Induced Membrane Damage and Is a Potential Target for beta-Lactam-Daptomycin Synergy. Antimicrob Agents Chemother **60**, 451-458, doi:10.1128/AAC.02071-15 (2016).
30 Lee, Y. H., Kingston, A. W. & Helmann, J. D. Glutamate dehydrogenase affects resistance to cell wall antibiotics in Bacillus subtilis. J Bacteriol **194**, 993-1001, doi:10.1128/JB.06547-11 (2012).
31 Singh, V. K. et al. Roles of pyruvate dehydrogenase and branched-chain alpha-keto acid dehydrogenase in branched-chain membrane fatty acid levels and associated functions in Staphylococcus aureus. J Med Microbiol **67**, 570-578, doi:10.1099/jmm.0.000707 (2018).
32 Reed, L. J. A trail of research from lipoic acid to alpha-keto acid dehydrogenase complexes. J Biol Chem **276**, 38329-38336, doi:10.1074/jbc.R100026200 (2001).
33 Packer, L., Witt, E. H. & Tritschler, H. J. alpha-Lipoic acid as a biological a ntioxidant. Free Radic Biol Med **19**, 227-250 (1995).
34 Piras, C. et al. Comparative proteomics to evaluate multi drug resistance in Escherichia coli. Mol Biosyst **8**, 1060-1067, doi:10.1039/c1mb05385j (2012).
35 Tsugawa, H. et al. Alpha-ketoglutarate oxidoreductase, an essential salvage enzyme of energy metabolism, in coccoid form of Helicobacter pylori. Biochem Biophys Res Commun **376**, 46-51, doi:10.1016/j.bbrc.2008.08.078 (2008).
36 Carlson-Banning, K. M. et al. Toward repurposing ciclopirox as an antibiotic against drug-resistant Acinetobacter baumannii, Escherichia coli, and Klebsiella pneumoniae. PLoS One **8**, e69646, doi:10.1371/journal.pone.0069646 (2013).
37 Parsons, J. B. & Rock, C. O. Bacterial lipids: metabolism and membrane homeostasis. Prog Lipid Res **52**, 249-276, doi:10.1016/j.plipres.2013.02.002 (2013).
38 Spector, M. P. et al. The medium-/long-chain fatty acyl-CoA dehydrogenase (fadF) gene of Salmonella typhimurium is a phase 1 starvation-stress response (SSR) locus. Microbiology **145** (Pt 1), 15-31, doi:10.1099/13500872-145-1-15 (1999).
39 Hamada, N., Watanabe, K., Arai, M., Hiramine, H. & Umemoto, T. Cytokine production induced by a 67-kDa fimbrial protein from Porphyromonas gingivalis. Oral Microbiol Immunol **17**, 197-200 (2002).
40 Enersen, M., Nakano, K. & Amano, A. Porphyromonas gingivalis fimbriae. J Oral Microbiol **5**, doi:10.3402/jom.v5i0.20265 (2013).
41 Csefalvay, E. et al. Functional coupling of duplex translocation to DNA cleavage in a type I restriction enzyme. PLoS One **10**, e0128700, doi:10.1371/journal.pone.0128700 (2015).
42 Chu, L., Bramanti, T. E., Ebersole, J. L. & Holt, S. C. Hemolytic activity in the periodontopathogen Porphyromonas gingivalis: kinetics of enzyme release and localization. Infect Immun **59**, 1932-1940 (1991).
43 Andersen, C. Channel-tunnels: outer membrane components of type I secretion systems and multidrug efflux pumps of Gram-negative bacteria. Rev Physiol Biochem Pharmacol **147**, 122-165, doi:10.1007/s10254-003-0008-y (2003).
44 Lima, T. B. et al. Bacterial resistance mechanism: what proteomics can elucidate. Faseb J **27**, 1291-1303, doi:10.1096/fj.12-221127 (2013).
45 Surekha, K. et al. Investigation of vital pathogenic target orotate phosphoribosyltransferases (OPRTase) from Thermus thermophilus HB8: Phylogenetic and molecular modeling approach. Gene **583**, 102-111, doi:10.1016/j.gene.2016.02.006 (2016).
46 Zhao, M., Wijayasinghe, Y. S., Bhansali, P., Viola, R. E. & Blumenthal, R. M. A surprising range of modified-methionyl S-adenosylmethionine analogues support bacterial growth. Microbiology **161**, 674-682, doi:10.1099/mic.0.000034 (2015).
47 Hosaka, T., Xu, J. & Ochi, K. Increased expression of ribosome recycling factor is responsible for the enhanced protein synthesis during the late growth phase in an antibiotic-overproducing Streptomyces coelicolor ribosomal rpsL mutant. Mol Microbiol **61**, 883-897, doi:10.1111/j.1365-2958.2006.05285.x (2006).
48 Breidenstein, E. B. et al. The Lon protease is essential for full virulence in Pseudomonas aeruginosa. PLoS One **7**, e49123, doi:10.1371/journal.pone.0049123 (2012).
49 Abby, S. S. et al. Identification of protein secretion systems in bacterial genomes. Sci Rep **6**, 23080, doi:10.1038/srep23080 (2016).

50 Salacha, R. et al. The Pseudomonas aeruginosa patatin-like protein PlpD is the archetype of a novel Type V secretion system. Environ Microbiol **12**, 1498-1512, doi:10.1111/j.1462-2920.2010.02174.x (2010).
51 Kang, J. et al. Effect of phosphoglucosamine mutase on biofilm formation and antimicrobial susceptibilities in M. smegmatis glmM gene knockdown strain. PLoS One **8**, e61589, doi:10.1371/journal.pone.0061589 (2013).
52 Grubman, A. et al. Vitamin B6 is required for full motility and virulence in Helicobacter pylori. MBio **1**, doi:10.1128/mBio.00112-10 (2010).
53 Dick, T., Manjunatha, U., Kappes, B. & Gengenbacher, M. Vitamin B6 biosynthesis is essential for survival and virulence of Mycobacterium tuberculosis. Mol Microbiol **78**, 980-988, doi:10.1111/j.1365-2958.2010.07381.x (2010).
54 Koskiniemi, S. et al. Rhs proteins from diverse bacteria mediate intercellular competition. Proc Natl Acad Sci U S A **110**, 7032-7037, doi:10.1073/pnas.1300627110 (2013).
55 Lee, S. E. et al. The pyrH gene of Vibrio vulnificus is an essential in vivo survival factor. Infect Immun **75**, 2795-2801, doi:10.1128/IAI.01499-06 (2007).

Supplementary Table S8 Overview of upregulated or uniquely present proteins in antiseptic-adapted *P. intermedia*

Adapted species	Protein name (accession number)	Protein function	
		Native	Associated with resistance/virulence
**CHX-adapted *Pi***	Elongation factor Tu ° (A0A1P8JJL2)	Protein biosynthesis	Mutations of the encoding gene increase resistance to antibiotics [1]
	D-3-phosphoglycerate dehydrogenase ° (A0A0H5BLP7)	Amino acid biosynthesis	Upregulated in presence of cell wall-active antibiotics [2]<br>Bacterial adhesion and invasion [3]
	Succinate--CoA ligase [ADP-forming] subunit beta ° (A0A0S3UJM7)	Participation in Krebs cycle	Expressed in antibiotic-resistant species [4]
	Cell envelope biogenesis protein OmpA ° (A0A246ET31)	Involved in membrane synthesis	Outer-membrane porin (ompA) associated with antibiotic resistance [5]
	Uncharacterized protein ° (A0A246ESS9)	Possible active segregation of plasmid copies prior to cell division	-
	Uncharacterized protein ° (A0A0S3UHY7)	Possible outer membrane protein 28	Immunogenic factors [6,7]
	Rubrerythrin ° (A0A246ESF3)	Involved in oxidative stress tolerance	Increases bacterial survival against host burst, contributing to the morbidity and mortality associated with infection [8]
	Lysine--tRNA ligase ° (A0A0S3UIQ9)	Participation in lysine biosynthesis	Increase in peptidoglycan synthesis [9]
	Dihydrolipoyl dehydrogenase ° (A0A0S3UHW5)	Plays a vital role in glycolysis, gluconeogenesis, Krebs cycle and cell redox homeostasis	Implicated in resistance to antimicrobial peptides [10]<br>Protects against oxidative stress [11]<br>Manipulates immune response [11]
	Uncharacterized protein ° (A0A246EWJ5)	Possible T9SS C-terminal target domain-containing protein	Involved in the secretion of virulence factors [12,13]<br>Cell wall biosynthesis [12]<br>Heme acquisition [13]
	Rod shape-determining protein MreB ° (A0A0S3ULS5)	Involved in formation of the rod shape of the cell	Peptidoglycan synthesis [14]
	Uncharacterized protein ***** (A0A246EXJ4)	No function found	-
**CPC-adapted *Pi***	Glyceraldehyde-3-phosphate dehydrogenase ° (A0A0S3UIF6)	Participation in glycolysis	Decreased susceptibility to antibiotics [15]<br>Immune system evasion [16]<br>Bacterial colonization [17]
	Uncharacterized protein ° (A0A0S3UHY7)	Possible outer membrane protein 28	Immunogenic factors [6,7]
	Uncharacterized protein ° (A0A246EWJ5)	Possible T9SS C-terminal target domain-containing protein	Involved in the secretion of virulence factors [12,13]<br>Cell wall biosynthesis [12]<br>Heme acquisition [13]
	Putative ribose-5-phosphate isomerase B ° (A0A0H5B761)	Involved in pentose-phosphate pathway	Increases survival via regulation of quorum sensing under stress conditions [18]
	UDP-glucose 4-epimerase ° (A0A1P8JJ56)	Galactose metabolism and glycoprotein or glycolipid synthesis	Absence of this gene reduces bacterial antibiotic resistance [19]<br>Participates also in the synthesis of lipopolysaccharides [19]
	2-oxoglutarate ferredoxin oxidoreductase subunit gamma ° (A0A0S3UI80)	Participation in Krebs cycle	Mutations of the encoding gene contribute to antibiotic resistance [20]

	Uncharacterized protein ***** (A0A246EXJ4)	No function found	-
	Uncharacterized protein ****** (A0A0S3UJG4)	Possible T9SS C-terminal target domain-containing protein	Involved in the secretion of virulence factors [12,13]<br>Cell wall biosynthesis [12]<br>Heme acquisition [13]

CHX-adapted *Pi*: *P. intermedia* exposed to chlorhexidine during 10 passages; CPC-adapted *Pi*: *P. intermedia* exposed to cetylpyridinium chloride during 10 passages.

° Proteins also detected in wild type *P. intermedia* but significantly upregulated in the adapted species (*P* < 0.05).

* Proteins uniquely present in both CHX-adapted and CPC-adapted *P. intermedia* compared to the wild type species.

** Proteins uniquely present in either CHX- or CPC-adapted *P. intermedia* compared to the wild type species.

References used in Supplementary Table S8

1 Cappellano, C., Monti, F., Sosio, M., Donadio, S. & Sarubbi, E. Natural kirromycin resistance of elongation factor Tu from the kirrothricin producer Streptomyces cinnamoneus. *Microbiology* **143 (Pt 2)**, 617-624, doi:10.1099/00221287-143-2-617 (1997).

2 Utaida, S. *et al.* Genome-wide transcriptional profiling of the response of Staphylococcus aureus to cell-wall-active antibiotics reveals a cell-wall-stress stimulon. *Microbiology* **149**, 2719-2732, doi:10.1099/mic.0.26426-0 (2003).

3 Yasuda, M. *et al.* Pseudomonas aeruginosa serA Gene Is Required for Bacterial Translocation through Caco-2 Cell Monolayers. *PLoS One* **12**, e0169367, doi:10.1371/journal.pone.0169367 (2017).

4 Neri, A. *et al.* Neisseria meningitidis rifampicin resistant strains: analysis of protein differentially expressed. *Bmc Microbiol* **10**, 246, doi:10.1186/1471-2180-10-246 (2010).

5 Smani, Y. *et al.* Role of OmpA in the multidrug resistance phenotype of Acinetobacter baumannii. *Antimicrob Agents Chemother* **58**, 1806-1808, doi:10.1128/AAC.02101-13 (2014).

6 Im, Y. B., Park, W. B., Jung, M., Kim, S. & Yoo, H. S. Comparative Analysis of Immune Responses to Outer Membrane Antigens OMP10, OMP19, and OMP28 of Brucella abortus. *Jpn J Infect Dis* **71**, 197-204, doi:10.7883/yoken.JJID.2017.019 (2018).

7 Neves-Ferreira, A. G. *et al.* Complete amino acid sequence and location of Omp-28, an important immunogenic protein from Salmonella enterica serovar typhi. *Protein J* **23**, 71-77 (2004).

8 Mydel, P. *et al.* Roles of the host oxidative immune response and bacterial antioxidant rubrerythrin during Porphyromonas gingivalis infection. *PLoS Pathog* **2**, e76, doi:10.1371/journal.ppat.0020076 (2006).

9 Dare, K. & Ibba, M. Roles of tRNA in cell wall biosynthesis. *Wiley Interdiscip Rev RNA* **3**, 247-264, doi:10.1002/wrna.1108 (2012).

10 Shen, C. J., Kuo, T. Y., Lin, C. C., Chow, L. P. & Chen, W. J. Proteomic identification of membrane proteins regulating antimicrobial peptide resistance in Vibrio parahaemolyticus. *J Appl Microbiol* **108**, 1398-1407, doi:10.1111/j.1365-2672.2009.04544.x (2010).

11 Smith, A. W., Roche, H., Trombe, M. C., Briles, D. E. & Hakansson, A. Characterization of the dihydrolipoamide dehydrogenase from Streptococcus pneumoniae and its role in pneumococcal infection. *Mol Microbiol* **44**, 431-448 (2002).

12 Gorasia, D. G. *et al.* Porphyromonas gingivalis Type IX Secretion Substrates Are Cleaved and Modified by a Sortase-Like Mechanism. *PLoS Pathog* **11**, e1005152, doi:10.1371/journal.ppat.1005152 (2015).

13 Lasica, A. M., Ksiazek, M., Madej, M. & Potempa, J. The Type IX Secretion System (T9SS): Highlights and Recent Insights into Its Structure and Function. *Front Cell Infect Microbiol* **7**, 215, doi:10.3389/fcimb.2017.00215 (2017).

14 Morgenstein, R. M. *et al.* RodZ links MreB to cell wall synthesis to mediate MreB rotation and robust morphogenesis. *Proc Natl Acad Sci U S A* **112**, 12510-12515, doi:10.1073/pnas.1509610112 (2015).

15 Fajardo, A. *et al.* The neglected intrinsic resistome of bacterial pathogens. *PLoS One* **3**, e1619, doi:10.1371/journal.pone.0001619 (2008).

16 Terao, Y., Yamaguchi, M., Hamada, S. & Kawabata, S. Multifunctional glyceraldehyde-3-phosphate dehydrogenase of Streptococcus pyogenes is essential for evasion from neutrophils. *J Biol Chem* **281**, 14215-14223, doi:10.1074/jbc.M513408200 (2006).

17 Kinoshita, H. *et al.* Cell surface Lactobacillus plantarum LA 318 glyceraldehyde-3-phosphate dehydrogenase (GAPDH) adheres to human colonic mucin. *J Appl Microbiol* **104**, 1667-1674, doi:10.1111/j.1365-2672.2007.03679.x (2008).

18 Yuan, L., Hillman, J. D. & Progulske-Fox, A. Microarray analysis of quorum-sensing-regulated genes in Porphyromonas gingivalis. *Infect Immun* **73**, 4146-4154, doi:10.1128/IAI.73.7.4146-4154.2005 (2005).

19 Carlson-Banning, K. M. *et al.* Toward repurposing ciclopirox as an antibiotic against drug-resistant Acinetobacter baumannii, Escherichia coli, and Klebsiella pneumoniae. *PLoS One* **8**, e69646, doi:10.1371/journal.pone.0069646 (2013).

20 Kaakoush, N. O., Asencio, C., Megraud, F. & Mendz, G. L. A redox basis for metronidazole resistance in Helicobacter pylori. *Antimicrob Agents Chemother* **53**, 1884-1891, doi:10.1128/AAC.01449-08 (2009).

Supplementary Table S9 Overview of downregulated or undetected proteins in antiseptic-adapted oral pathogens

CHX-adapted *A. actinomycetemcomitans*<br>Protein name (accession number)	CPC-adapted *A. actinomycetemcomitans*<br>Protein name (accession number)
Pyridoxal kinase PdxY ° (A0A142FY88)	Pyridoxal kinase PdxY ° (A0A142FY88)
DNA-binding protein Fis ° (A0A142G0Y8)	Ferric iron binding protein ° (O32480)
Protein HflC ° (A0A142FYR3)	FAD:protein FMN transferase ° (A0A142FZX5)
Cysteine desulfurase IscS ° (A0A142G1Z3)	Probable transcriptional regulatory protein ° (A0A142G241)
Glycine--tRNA ligase alpha subunit ° (A0A142G0R7)	Ribose-phosphate pyrophosphokinase ° (A0A142FYA4)
Oxidoreductase ° (A0A142G1L7)	50S ribosomal protein L23 ° (P55839)
Glycerol-3-phosphate acyltransferase ° (A0A142FZH6)	Phosphofructokinase ° (A0A142G1J4)
HAD family hydrolase ° (A0A142G1L0)	Iron-sulfur cluster assembly scaffold protein IscU ° (A0A142G1Z2)
HemX protein ° (A0A142FZT0)	Arginine ABC transporter substrate-binding protein ° (A0A142G1G5)
Pyruvate dehydrogenase E1 component * (A0A142FXW4)	Orotate phosphoribosyltransferase ° (A0A142FZZ3)
Protein TadE * (Q7X0L4)	RNA polymerase sigma factor RpoD ° (A0A142FZ58)
Na(+)-translocating NADH-quinone reductase subunit A * (A0A142FZX6)	ATP-dependent RNA helicase DeaD ° (A0A142G129)
OmpA-like outer membrane protein * (Q9S5J9)	Trimethylamine N-oxide reductase I catalytic subunit ° (A0A142FXF9)
Peptidoglycan-associated lipoprotein * (I6LEK5)	Dimethyl sulfoxide reductase ° (A0A142G0S2)
Dihydrolipoyl dehydrogenase * (A0A142FXW3)	Maltose/maltodextrin import ATP-binding protein MalK ° (A0A142G2Z0)
OxyR like protein ** (Q75TM5)	Glutaredoxin ° (Q9ZAB9)
	30S ribosomal protein S8 * (A0A142FZB9)
	30S ribosomal protein S20 * (A0A142FZ42)
	Thioredoxin reductase * (A0A142G2J2)
	Integration host factor subunit alpha * (A0A142G363)
	Phosphoenolpyruvate carboxykinase (ATP) * (A0A142FZT8)
	OxyR like protein ** (Q75TM5)

CHX-adapted *F. nucleatum*<br>Protein name (accession number)	CPC-adapted *F. nucleatum*<br>Protein name (accession number)
Glycerol-3-phosphate dehydrogenase ° (A5TSZ7)	D-lactate dehydrogenase ° (A5TXC5)
50S ribosomal protein L6 ° (A5TVC2)	Propanediol dehydratase small subunit ° (A5TRI7)
DNA-directed RNA polymerase subunit beta ° (A5TS57)	DNA gyrase subunit A ° (A5TSE0)
Tyrosine phenol-lyase ° (A5TXB5)	30S ribosomal protein S13 * (A5TTT0)
Galactokinase ° (A5TSD1)	Butyryl-CoA dehydrogenase * (A5TRL6)
Uncharacterized protein ° (A5TV54)	Enolase * (A5TVQ3)
Threonine ammonia-lyase ° (A5TY53)	Adenylosuccinate lyase * (A5TTK8)
Glucose-1-phosphate adenylyltransferase ° (A5TWP0)	Cation ABC superfamily ATP binding cassette transporter binding protein * (A5TVU1)
Glycerol kinase ° (A5TRJ8)	Tyrosine phenol-lyase * (A5TXB5)
DNA gyrase subunit A ° (A5TSE0)	Malate dehydrogenase ** (A5TTZ6)
Protein-N(Pi)-phosphohistidine--sugar phosphotransferase ° (A5TUY6)	Adenine phosphoribosyltransferase ** (A5TS8)
Methionine--tRNA ligase ° (A5TTU7)	Uncharacterized protein ** (A5TU42)
M42 family glucanase/peptidase ° (A5TUQ0)	
Glucose-1-phosphate adenylyltransferase * (A5TWP0)	
Endoribonuclease translation inhibitor * (A5TRY8)	
Adenine phosphoribosyltransferase ** (A5TS8)	
Malate dehydrogenase ** (A5TTZ6)	
Uncharacterized protein ** (A5TU42)	

**CHX-adapted *P. gingivalis*** **Protein name (accession number)**	**CPC-adapted *P. gingivalis*** **Protein name (accession number)**
Alpha-L-fucosidase ° (A0A1R4DVQ8)	Protein TonB ° (A0A1R4DTE1)
Malate dehydrogenase ° (A0A254N187)	Signal peptidase I ° (A0A1R4DZE0)
Peptidase M13 ° (A0A1R4DWD8)	Aspartate--tRNA ligase ° (A0A1R4DRA5)
Capsule biosynthesis protein CapM ° (A0A254MU12)	Malate dehydrogenase ° (A0A254N187)
Ribonuclease R ° (A0A254MTG1)	Peptidase M13 ° (A0A1R4DWD8)
Lipoprotein ° (A0A1R4AG27)	Uncharacterized protein ° (A0A1R4DQW7)
Phosphoglucomutase * (A0A1R4AEN0)	Aminopeptidase ° (A0A1R4DXN6)
Enoyl-CoA hydratase * (A0A1R4AEV7)	4-hydroxy-3-methylbut-2-en-1-yl diphosphate synthase (flavodoxin) ° (A0A1R4AG43)
MEGL/2-ABD * (C3VMV9)	Thiol reductase thioredoxin ° (A0A134DNP8)
RagB protein * (Q4KTT0)	Capsule biosynthesis protein CapM ° (A0A254MU12)
2-oxoglutarate ferredoxin oxidoreductase subunitgamma * (A0A1R4DQ72)	Proline--tRNA ligase ° (A0A134DQ07)
Peptidase S10 * (A0A1R4DXH1)q	Uncharacterized protein ° (A0A134DMW0)
Transketolase * (A0A1R4AHF2)	Phosphoribosylformylglycinamidine cyclo-ligase ° (A0A1R4DUK5)
Membrane protein * (A0A1R4DSZ6)	Phosphoribosylformylglycinamidine synthase ° (A0A1R4ADZ6)
	Uncharacterized protein ° (A0A1R4AFX0)
	NAD metabolism ATPase/kinase ° (A0A1R4AFI3)
	Pyridoxine 5'-phosphate synthase (Fragment) ° (Q7WZB3)
	ATP-dependent Clp protease proteolytic subunit ° (A0A1R4DU46)
	Alpha-amylase ° (A0A1R4AFV6)
	Ribonuclease R ° (A0A254MTG1)
	Aspartate-semialdehyde dehydrogenase ° (A0A1R4AFS7)
	Pseudouridine synthase ° (A0A254MSH9)
	Peroxiredoxin * (A0A254MTP1)
	FadL family T9SS component outer membrane protein PorV * (A0A134DMH4)
	RND transporter MFP subunit * (A0A1R4AD39)
	Branched-chain amino acid dehydrogenase * (A0A254N2U4)
	Uncharacterized protein * (A0A1R4DV80)
	DNA gyrase subunit A * (A0A142FXK6)
	Oxaloacetate decarboxylase * (A0A1R4AHI2)
	Acyl-[acyl-carrier-protein]--UDP-N-acetylglucosamine O-acyltransferase * (A0A1R4DUU0)
	Thiol:disulfide interchange protein * (A0A134DRC7)
	Orotidine 5'-phosphate decarboxylase * (A0A254MZL4)
	Polyribonucleotide nucleotidyltransferase * (A0A142G3C3)
	Uncharacterized protein * (A0A134DNU1)
	Bifunctional purine biosynthesis protein PurH * (A0A1R4AH02)
	Carbonic anhydrase * (A0A1R4DU91)
	Chaperonin GroL * (A0A254N4Z3)
	Membrane protein * (A0A1R4DSZ6)

**CHX-adapted *P. intermedia*** **Protein name (accession number)**	**CPC-adapted *P. intermedia*** **Protein name (accession number)**
50S ribosomal protein ° (A0A0S3UJA6)	Peptidyl-dipeptidase ° (A0A0S3UP02)
Peptidyl-dipeptidase ° (A0A0S3UP02)	Polysaccharide biosynthesis/export protein ° (A0A0S3UGE9)
Phosphate acetyltransferase ° (A0A0S3UJK1)	Uncharacterized protein ° (A0A0S3UK47)
30S ribosomal protein S15 ° (A0A0T7ANA3)	L-asparaginase ° (A0A0S3ULE3)
50S ribosomal protein L16 ° (A0A0H5B2S9)	Nuclease ° (A0A140JTF2)

AMP-binding enzyme ° (A0A0H5B7E6)	Meso-diaminopimelate D-dehydrogenase ° (A0A0S3UJ72)
Enoyl-[acyl-carrier-protein] reductase [NADH] ° (A0A1P8JJZ2)	Uncharacterized protein ° (A0A0S3UIQ4)
Hsp20/alpha crystallin family protein ° (A0A0S3UIN8)	S9 family peptidase ° (A0A1P8JLL4)
Polysaccharide biosynthesis/export protein ° (A0A0S3UGE9)	Formate C-acetyltransferase * (A0A246EUW1)
3-oxoacyl-[acyl-carrier-protein] synthase 2 ° (A0A0S3UK56)	Fructanase * (C8KIP0)
DNA gyrase subunit B ° (A0A246EV89)	Tyrosine-protein kinase * (A0A0S3UGA7)
Peptidase M13 ° (A0A1P8JL86)	AMP-binding enzyme * (A0A0H5B7E6)
2,3-bisphosphoglycerate-independent phosphoglycerate mutase ° (A0A0S3UKU6)	Phosphate acetyltransferase * (A0A0S3UJK1)
50S ribosomal protein L19 ° (A0A0S3UJ76)	Phosphoenolpyruvate carboxykinase (ATP) * (A0A0S3UL62)
Uncharacterized protein ° (A0A0S3UK47)	50S ribosomal protein L15 * (A0A0S3UJF0)
Phosphorylase ° (A0A0H5B6V0)	Phosphorylase * (A0A0H5B6V0)
Peptidyl-prolyl cis-trans isomerase ° (A0A0T7APV5)	AcrA/AcrE family multidrug resistance protein *
Polyribonucleotide nucleotidyltransferase ° (A0A246EVG1)	4-alpha-glucanotransferase * (A0A0S3UIA9)
50S ribosomal protein L28 ° (A0A0H5BMT4)	Pyruvate carboxylase subunit B * (A0A0S3UIU0)
Aminoacyl-histidine dipeptidase ° (A0A0T7ANJ3)	Metalloendopeptidase PepO ** (A0A0S3UJX5)
50S ribosomal protein L3 ° (A0A0S3UJT6)	50S ribosomal protein L11 ** (A0A0H5B2V0)
30S ribosomal protein S3 ° (A0A0S3UJE4)	
AcrA/AcrE family multidrug resistance protein ° (A0A0T7AN94)	
Acyl-CoA dehydrogenase ° (A0A0S3UIQ2)	
Electron transfer flavoprotein alpha subunit ° (A0A0S3UIQ7)	
Methionine--tRNA ligase ° (A0A0S3UJL4)	
Transcriptional regulator ° (A0A0S3UHX2)	
50S ribosomal protein L24 1 ° (A0A0S3UJ97)	
L-asparaginase ° (A0A140JTF2)	
Nuclease ° (A0A140JTF2)	
DNA topoisomerase 1 ° (A0A0S3UKG6)	
M16 family peptidase ° (A0A0S3UM04)	
Biopolymer transporter ExbD ° (A0A0T7AN51)	
Isoleucine--tRNA ligase ° (A0A0T7AP38)	
Meso-diaminopimelate D-dehydrogenase ° (A0A0S3UJ72)	
50S ribosomal protein L18 ° (A0A246EWL1)	
Peptidyl-prolyl cis-trans isomerase ° (A0A0T7APV5)	
30S ribosomal protein S10 ° (A0A0H5B636)	
50S ribosomal protein L23 ° (A0A0S3UJA9)	
Uncharacterized protein ° (A0A0S3UIQ4)	
30S ribosomal protein S6 ° (A0A0H5BL28)	
30S ribosomal protein S4 * (A0A0H5BM77)	
50S ribosomal protein L4 * (A0A0S3UJA6)	
Succinate dehydrogenase * (A0A246EVV6)	
50S ribosomal protein L13 * (A0A0H5AZI5)	
Bifunctional protein FolD * (A0A0S3UI34)	
30S ribosomal protein S7 * (A0A0S3UJC6)	
Carboxyl transferase * (A0A0S3UHW1)	
Thiol:disulfide interchange protein DsbD * (A0A0T7AN77)	
Metalloendopeptidase PepO ** (A0A0S3UJX5)	
50S ribosomal protein L11 ** (A0A0H5B2V0)	

CHX-adapted: bacteria exposed to chlorhexidine during 10 passages; CPC-adapted: bacteria exposed to cetylpyridinium chloride during 10 passages.

° Proteins not detected in the antiseptic-adapted species compared to the wild type species

* Proteins significantly downregulated in either CHX- or CPC-adapted species compared to the wild type species ($P < 0.05$).

** Proteins significantly downregulated in both CHX- and CPC-adapted species compared to the wild type species ($P < 0.05$).
